# Regional patterns of global industrial energy demands as a foundation for modelling decarbonization pathways

**DOI:** 10.1038/s41597-025-06206-y

**Published:** 2025-11-05

**Authors:** Anđelka Kerekeš, Arne Burdack, Ganesh Deepak Rupakula, Felix Lippkau, Heidi Heinrichs, Philipp Kuhn, Detlef Stolten, Jochen Linßen, Markus Blesl, Thomas Hamacher

**Affiliations:** 1 Technical University of Munich (TUM), TUM School of Engineering and Design, Department of Energy and Process Engineering, 85748 Garching b, München, Germany; 2 Institute of Climate and Energy Systems – Jülich Systems Analysis (ICE-2), Forschungszentrum Jülich, 52425 Jülich, Germany; 3 RWTH Aachen University, Chair for Fuel Cells, Faculty of Mechanical Engineering, Aachen, 52062 Germany; 4 Institute of Energy Economics and Rational Energy Use (IER), University of Stuttgart, 70565 Stuttgart, Germany; 5 University of Siegen, Department of Mechanical Engineering, 57076 Siegen, Germany

**Keywords:** Energy efficiency, Energy supply and demand

## Abstract

Decarbonisation of the industry sector is particularly challenging given its huge energy demand and high complexity of its various production processes. Additionally, regional differences in energy demand patterns affect the possible transition pathways. This study assesses energy demands of the five most energy-intensive industries iron and steel, non-ferrous metals, non-metallic minerals, pulp and paper, and chemicals for 2018, the most recent year not affected by the Covid-19 pandemic for 16 exemplary world regions of the TIAM global energy system model. This new approach follows three steps: mapping industrial production quantities, determining theoretical specific energy demands by applying the best available technologies and theoretical minima, and calculating inefficiency of the energy usage based on the International Energy Agency’s energy balances. For the first time, this study provides regional inefficiency factors for the energy-intensive industrial subsectors globally based on a consistent methodology. It diversifies between electricity, different fuels and chemical feedstock demands. The resulting dataset is meant for energy system modellers and decision-makers to analyse industrial energy demand and develop decarbonization strategies.

## Background & Summary

Industry is one of the biggest energy demand sectors with far ranging impacts on society, economy and environment making its decarbonization a pressing challenge. Decarbonization strategies in industry are heavily dependent on measures like energy efficiency improvements, fuel shifts or changing of the process routes^1^. However, there is a lack of recent, sufficiently detailed and openly available, globally consistent data on the current state of these measures, which further hampers the reliable development and implementation of decarbonization strategies.

The existing literature predominantly addresses process technologies for the production of a couple of main products within a specific industrial subsector. This trend is particularly pronounced in the chemical industry^2,3^. Certain scientific reports provide broader overviews within an industrial subsector. The International Energy Agency (IEA) study regarding chemical and petrochemical sector^4^ offers an extensive overview of chemical production processes at the time of its publication, making it a valuable reference for energy system modelling. However, it does not account for newer processes introduced in recent years. Additionally, while the study discusses process efficiency and energy data reliability for some world regions, it does not discuss the geographical distribution of the production processes itself. Dechema’s research^5^ describes both current-state as well as emerging low-carbon chemical and petrochemical production processes in a structured manner, facilitating also their incorporation into energy system models. Consideration of current-state processes is orientated on technologies common for European market. Although IEA’s study on future of petrochemicals^6^ does not quantify all inputs and outputs of the main chemical and petrochemical products, it examines the regional differences in intensity of the energy usage and the feedstock for their production. It also compares production data of elaborated products and world regions for a selected historical year and future projections. In the cement industry, IEA models cement manufacturing processes but in its technology roadmap^7^ it does not explicitly outline all necessary underlying assumptions. In contrast to previous studies, the CircEUlar project is more industry-spanning. It outlines, among others, production processes for main products of energy-intensive industries in order to model their life cycle^8^. This project, as well as some other existing models such as IndustryPlan and FORECAST, provides valuable data on the techno-economic assumptions of the industrial sector^8,9^. However, it does not comprehensively cover all industrial subsectors, excluding, for instance, process descriptions of copper, glass or pulp and paper. Moreover, these models primarily present globally available processes without reviewing their regional variations in current industrial plant stocks^9^. Hasanbeigi *et al*. emphasise the importance of considering different process routes when estimating regional specific energy demands of the iron and steel industry^10,11^. Similarly, Wang *et al*. propose a globally applicable method for quantifying energy consumption using an extended input-output analysis, yet their approach lacks detailed consideration of efficiency variations within processes^12^. Although these studies demonstrate that process efficiency can vary significantly, none of them offer a methodology that can be applied consistently on a global scale to determine specific energy demands and energy inefficiencies on a regional level.

Next, a similar pattern as seen for technology descriptions also applies to production quantities, where data availability tends to be subsector-specific and access to a comprehensive industry-spanning dataset remains restricted. The IEA maintains a collection of relevant production related data^13^, but its database is not publicly available. Eurostat compiles production quantities^14^, but it is limited to European countries. The *endemo* model integrates both process descriptions with associated energy requirements, as well as production quantity figures across all energy-intensive industrial subsectors^15^. However, its scope is also confined to European countries. U.S. Geological Survey (USGS)^16^ encompasses production quantities of metals and minerals for all world countries, giving thus a valuable data collection for modelling industry in energy systems. Yet, production quantities of non-metallic or non-mineral products such as chemicals and petrochemicals, pulp and paper, are out of its scope. Moreover, for some products like glass or for most of the chemicals and petrochemicals, no comprehensive and publicly available data sets were found.

Thus, identified challenges in literature include rather subsector-focussed technology descriptions and limited data collections. Furthermore, regional differences in energy consumption of production processes are not commonly explored. According to the literature reviewed above, only few studies compare energy demand values derived from their proposed specific energy consumption metrics with actual statistical data, which raises uncertainties about their applicability and validation in different geographical contexts. In conclusion, no publicly accessible global database integrating production quantities and technology data across all industrial subsectors while also accounting for regional differences in energy consumption and technological process variations was found. This gap presents a significant limitation for energy system modelling including industrial process analysis.

The goal of this study is therefore to provide openly available and consistent dataset containing information on production quantities and specific energy demands of energy intensive industrial subsectors world-wide, considering their spatial differences and based on the recent past data. Thus, this study aims to develop a consistent methodology for estimating regional energy demands of industrial processes on a global scale.

**Fig. 1:**
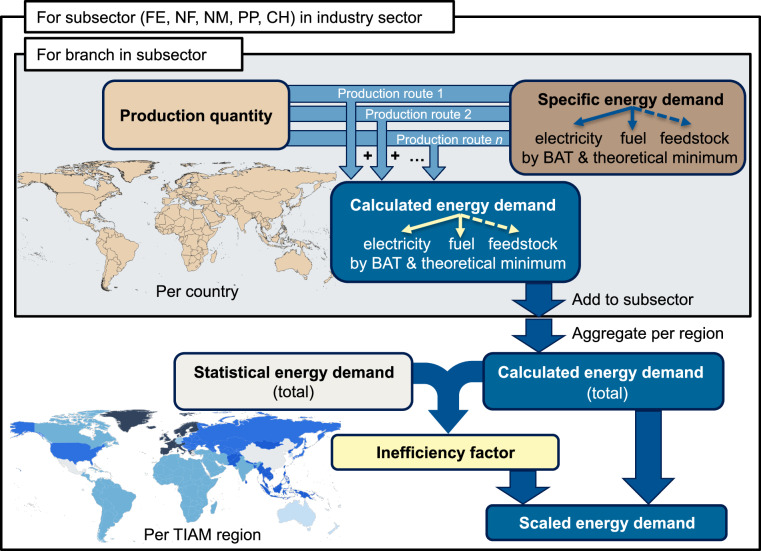
Workflow of the study. Industrial subsectors: Iron and steel (IS), Non-ferrous metals (NF), Non-metallic minerals (NM), Pulp and paper (PP), Chemical and petrochemical (CH).

More precisely, this study derives, in the first step, national production quantities on a global scale for the process routes of the five most energy-intensive industrial subsectors, namely iron and steel, non-ferrous metals, non-metallic minerals, pulp and paper, and chemical and petrochemical. In the second step, it defines the best available technology (BAT) for each process route of each industrial subsector and derives the respective specific energy demands. To make the dataset as widely applicable as possible, the specific energy demand is also equipped with the theoretical minimum energy demand. Third step encompasses the calculation of energy demands based on BAT and theoretical minimum and their comparison with the International Energy Agency’s (IEA) energy balances. The comparison is done per industrial subsector across the 16 aggregated TIAM^17,18^ world regions as a typical example for spatial resolution of global energy system models. In this way, the study identifies inefficiencies of the energy usage, whereby we define inefficiency factor as the ratio between the (real) statistical energy consumption and the minimal energy demand estimated for the BAT or theoretical minimum. The workflow of the study is given in the Fig. 1. As a most recent year for which data are available and are not affected by the disturbances of the Covid-19 pandemia, the year 2018 is taken as the reference year for which all the data are processed. Our underlying method and final data comply with the FAIR principles (**F**indability, **A**ccessibility, **I**nteroperability, and **R**euse)^19^.

Finally, the elaborated dataset can be a basis for future industrial energy demand estimations and potential energy reduction analysis. It represents a reference starting point for global projections which consider current particularities of energy-intensive industrial subsectors spatially resolved. The purpose of the inefficiency factors is to compare the different levels of efficiency across world regions and subsectors. At the same time, they have a balancing effect on the calculated total energy demand of regions where the processes included in the bottom-up calculations are not as representative of the entire sub-sector as in most other regions. The dataset can be furthermore used as an input for the industrial energy supply optimization. In this way, the study supports the energy system modelling community and the decision makers in their planning of adequate decarbonisation measures. Moreover, the study elucidates the available industry related data, and methods to overcome the existing data gaps.

## Methods

The study consists of three steps described in more detail in the following subsections. Initially, national production quantities are derived for a predefined set of process routes (step 1). These process routes cover those most relevant within the five most energy demanding industrial subsectors iron & steel, non-ferrous metals, non-metallic minerals, pulp & paper, and chemicals. If no differentiation between process routes was possible due to a lack of data, a standard process route was assumed. Next, for each process route, a global BAT and its specific energy demands for fuels, feedstock and electricity were derived from literature (step 2). In this step, each process route was additionally equipped with theoretical minimal specific energy determination. The theoretical minimal energy demand represents the physical lower boundary of energy consumption, based on stoichiometric and thermodynamic limits. To finally derive inefficiency factors (step 3), we utilize the IEA energy balance statistics^13^. As this database is proprietary and only contains industrial subsectors, we aggregated our results to energy demands of the five industrial subsectors within 16 world regions. The spatial resolution of the 16 world regions was taken from TIAM as a typical example of a global energy system model. This step was accompanied by a thorough plausibility check of our obtained results. Table 1 summarizes the covered industrial subsectors, branches and products within this study. The selection of the products has two main reasons. First reason is the specific energy demand attributable to a product depending on the processing stages it went through. For example, pyrometallurgical copper production route fabricates copper of rising purity from one processing stage to another. It is usual for production sites to fabricate intermediate products which did not undergo all of the processing stages as their final product. To calculate the right energy demand of the site, it is necessary to distinguish between the products. Second reason for the products selection results from the diversity of the possible products, especially in chemical sector. Thus, we concentrate on the products with highest energy requirements within the industrial branch.

**Table 1:** Structure of the elaborated 5 industrial subsectors with their 13 branches and 27 products.

Subsector	Branch	Products
Iron and Steel	Iron and steel	Primary steel
		Secondary steel
Non-ferrous metals	Aluminium	Primary aluminium
		Secondary aluminium
	Copper	Primary and secondary: copper matte (ca. 65% metal purity), refined copper (99.95% −99.99% metal purity)
Non-metallic minerals	Cement	Cement
	Glass	Container glass
		Flat glass
	Lime	Lime
Pulp and paper	Pulp	Chemical pulp
		Mechanical pulp
		Recycled pulp
	Paper	Graphical paper
		Hygiene paper
		Packaging paper
		Technical packaging paper
Chemical and petrochemical	Chlorine	Chlorine
	Methanol	Methanol
	Ammonia	Ammonia
	Olefins	Ethylene, propylene
	Aromatics	Benzene, toluene, (o-, p-, m-) xylene

### Step 1: National production quantities per process route

The production quantities of all the industrial products within each process route are derived for the year 2018. For each industrial subsector different data sources are utilized.

#### Iron & steel

**Fig. 2:**
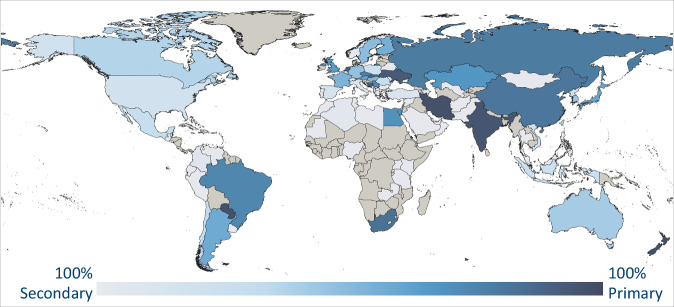
Primary and secondary steel shares in 2018. Grey stands for absence of steel production. Data source:^21^.

Steel is one of the best recyclable materials of the world^20^. In this study, recycled steel is called secondary steel, while steel made from iron ore is called primary steel. Primary steel is produced in two steps: First, iron ore is reduced to iron, then the iron is converted to steel by adjusting the carbon content and alloys^20^. The specific energy demand to produce primary and secondary steel, as well as between the primary steel routes, is very different (see step 2), so regional assumptions are made for the distribution of production among the routes. To obtain process route-specific national steel production quantities, the data of the US Geological Survey (USGS) are applied^21^. The USGS provides annual national production quantities of direct reduced iron (DRI), of pig iron (PI) and of steel separately. Primary steel production is assumed to be equivalent to the total iron production (sum of DRI and PI) because iron is by far the main input to steel, as no scrap addition is assumed^20^. Secondary steel production is consequently the difference between the steel and the primary steel. In reality, the primary production of steel from iron ore and the secondary production of steel from scrap are partly mixed, as steel scrap is added to the primary processes^20^. However, this study mathematically separates primary from secondary production to be able to assign a clear process route to secondary steel. The resulting spatially resolved shares of primary and secondary steel in total steel production are shown in Fig. 2.

#### Non-ferrous metals

Non-ferrous metals mainly include aluminium, copper, zinc, lead, nickel, tin and precious metals. As main representatives regarding energy intensity and green-house gas (GHG) emissions, aluminium and copper producing industries are elaborated within this study.

*Aluminium* production diversifies between the primary and secondary route, described in subsection Step 2: Specific energy demand for BAT and theoretical minimum. Production quantity of primary aluminium in 2018 comes from USGS Mineral yearbook^16^. Comprehensive secondary aluminium production quantities were not found for 2018 or any recent year but only for 2006^22^. Where possible more recent data were added: China^23^, Korea^24^, Germany^25^ and United States of America (USA)^26^ for 2018. For Brazil, in absence of any additional data, secondary aluminium is assumed to equal the recycled aluminium from beverage cans in 2023^27^. The updated values cover 54% of the global production in year 2018 estimated by the International Aluminium Institute (https://alucycle.international-aluminium.org/public-access/public-global-cycle/). Thus, production quantities for countries with 2006 data are scaled to account for the remaining 46% of the global production, ensuring a 100% global production coverage.

*Copper* can be produced by a pyrometallurgical or hydrometallurgical route, depending on the utilized ore. Primary copper is produced by both routes, whereas for secondary copper the pyrometallurgical route is more common. Moreover, copper undergoes multiple processing steps in the pyrometallurgical route which increase the purity of the obtained product. Copper obtained after the smelting has purity of around 65%^25^, whereas refined copper which undergoes the whole production process reaches 99.95–99.99% purity^25,28^. Each of these processing steps is accompanied with different energy demands. Therefore, to be able to correctly estimate the energy demand for the copper industry, we diversify between production amounts of primary and secondary copper from smelters, refined primary and secondary copper, as well as primary copper from hydrometallurgical route^16^. All mentioned production quantities for 2018 come from USGS Mineral yearbook^16^.

#### Non-metallic minerals

The non-metallic mineral products include ceramics, lime, glass, and cement. This paper focuses specifically on the production and energy demand of the lime, glass, and cement industries, as these sectors account for around 90% of the energy demand within the non-metallic minerals subsector based on our calculations with BAT routes and IEA statistics^13^. To capture the full energy demand of the non-metallic minerals subsector, “*other minerals*” are included in such a way that they account for 10% of the subsector’s total energy demand.

For the case of *cement*, a global database consisting of cement plants, locations and the total production capacities was provided in the Global database of cement production assets^29^. The total cement production quantities for each country for the years 1990–2021 were provided in another database^30^. Production values are taken from this database for the year 2018.

A publicly accessible source that provides global data on *glass* production does not currently exist. Similarly, national statistics are not available. Only the global glass production capacity of 217 million tonnes per year in 2018 is available (https://plants.glassglobal.com/). Consequently, a novel approach was devised to derive the national glass production. Soda ash is a crucial input for the glass industry^31^, with the glass industry accounting for around 50%^32^ of its total demand globally. The national demand for soda ash is therefore used as an indicator for the national glass demand. The national soda ash demands are calculated by summing up its national production^32^ and import^33^ and subtracting its national exports^33^. Shares of the national soda ash demands in the global demand are utilized to distribute the global glass capacity on a national level. Subsequently, in order to work with production quantities instead of capacities, national glass capacities are scaled with an assumed utilization rate of 90%, taking into account maintenance work and machine start-up times, to finally derive the national glass production in 2018.

National *lime* production in 2018 was sourced from the USGS annual publication^34^.

#### Pulp and paper

**Fig. 3:**
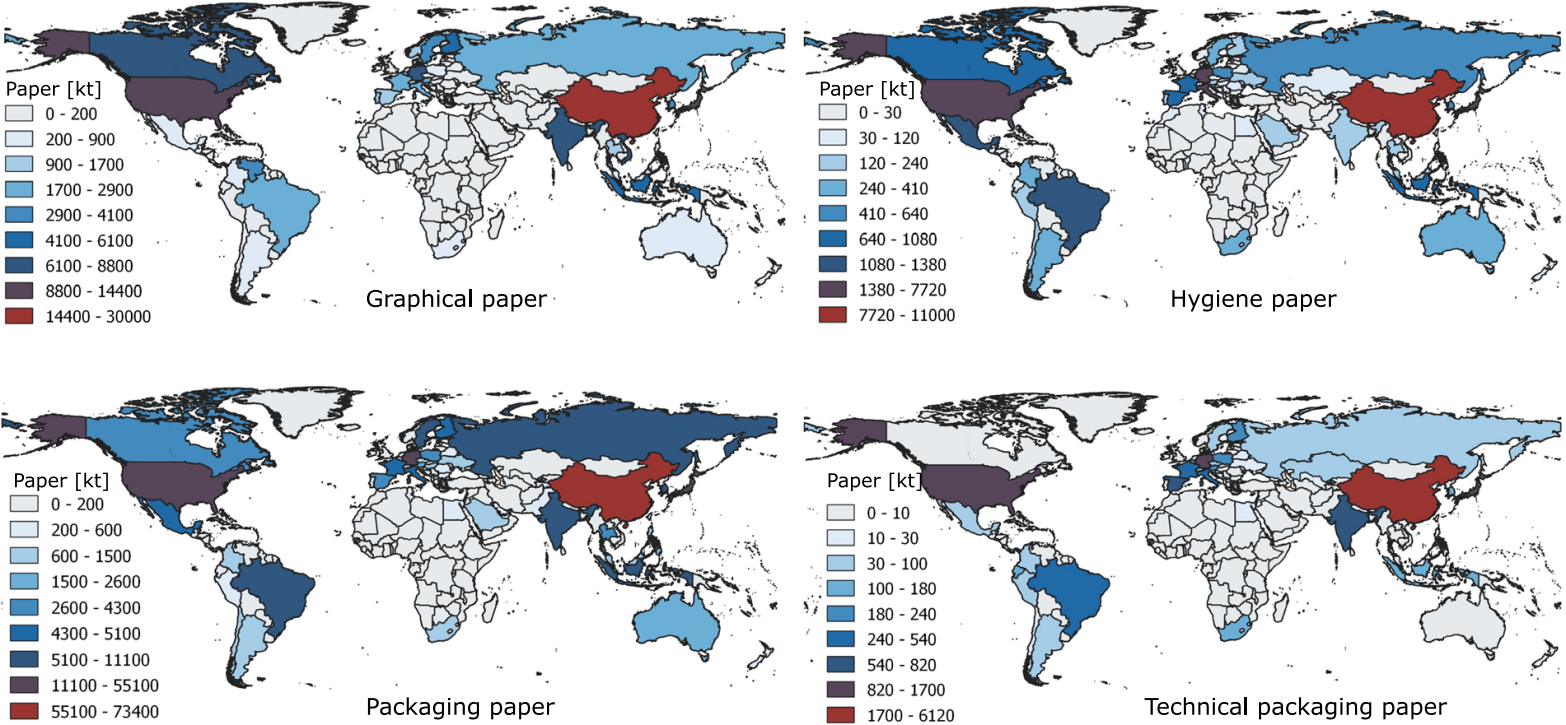
Paper production by category per country 2018. Data source:^35^.

*Pulp* is an intermediate product, which is used to produce paper and paperboard products. It consists primarily of cellulose fibres derived mostly from wood and other plant sources. Based on the manufacturing process, pulp on the market is generally categorized into chemical, mechanical and recycling pulp. Each pulping process has different electricity and fuel demand. The material input for chemical and mechanical pulp is typically wood, but in the case of recycled pulp it is waste or recycled paper. Production quantities of these three grades of pulp per country are provided in FAO Stat^35^. Similar to pulp, *paper* is categorized into four main categories, depending on the application and the manufacturing process. These include graphical, hygiene, packaging and technical packaging paper. Each grade of paper has different energy demand and specific pulp grade as the material input for the manufacture. Production quantities for all four grades of paper per country are provided in FAO Stat^35^ as well, and depicted in Fig. 3.

#### Chemical and petrochemical

The chemical industry is the most energy intensive of all industrial subsectors^6^. Among all the chemicals produced, five chemicals are responsible for about 70% of the total energy demand in this subsector^6^. These include ammonia, methanol, olefins, aromatics and chlorine. The production processes of these chemicals are provided in Table 1.

*Methanol* is an important intermediate chemical which is used as feedstock for production of various chemicals including formaldehyde, acetic acid and methyl-tertiary butyl ether. In 2018, 78 Mt of methanol was produced worldwide^36^. China dominates the methanol production with over 40% share globally^36^. To the author’s knowledge, a complete database consisting of methanol production country-wise does not exist. However, production quantities for China, Middle East, South America, North America, West Europe, Former Soviet Union, Southeast Asia and other TIAM regions were reported by the Methanol institute for the year 2018^36^. Production quantity for USA was represented in Statista^37^ and German production quantity was reported by the German chemical industry association for 2018^38^. From these references, production quantities of methanol are derived for each TIAM region.

*Chlorine* is another crucial chemical in the industry, with the majority of its production used in the manufacture of Polyvinyl Chloride (PVC) pipes^39,40^. The chlorine market has experienced consistent growth, increasing from 81 million tonnes of production in 2018 to 97.33 million tonnes in 2022^41^. Country wise capacity volumes for 2018 were taken from a detailed report containing production capacities of chlorine plants worldwide^39,40^. Production volumes of countries with high production quantity like USA^42^ and Germany were available for 2018^38^. The percentage of global chlorine production attributed to China was taken from another resource for 2018^43^. Based on these limited literature resources, a percentage contribution of TIAM regions with low production quantity that was not stated was calculated based on the capacity shares. The chlorine production volumes for each TIAM region are then derived based on the global production volume for the base year 2018.

Approximately 85% of today’s *ammonia* is used to produce nitrogen fertilizer^44^. The global production of ammonia is derived from the Nitrogen Statistics and Information of the USGS^45^. It is stated that nitrogen in this statistic is always converted to ammonia as a first step, before it is further converted to products based on ammonia^45^. By considering the molecular weight of ammonia and the contained nitrogen, where 1.21 tons of ammonia contain 1 ton of nitrogen, the ammonia production is derived.

Among o*lefins*, light olefins ethylene and propylene are most widely used, for example, in the manufacturing chain for plastics production^6^. They are the most-produced olefins globally with a production capacity of 185 and 120 million tonnes in 2018, respectively, and a capacity increase till 2022 of additional 70 million tonnes in total^46,47^. Next important olefin butadiene has a global production capacity of only about 20 million tonnes^48^. Therefore, under olefins this study elaborates ethylene and propylene. A comprehensive and up to date publicly available database for olefine production quantities was not found. Most comprehensive world-wide *ethylene* production capacity database found considers steam cracking capacities for ethylene production for 2008^49^. For ethylene capacity of European countries data were retrieved for 2021 (https://www.petrochemistry.eu/about-petrochemistry/petrochemicals-facts-and-figures/cracker-capacity). Capacity data for 2018 were available for China (https://www.icis.com/asian-chemical-connections/2023/03/global-oversupply-of-petrochemicals-to-hit-218m-tonnes-in-2023-the-highest-in-any-year-since-1990/), whereas for USA^50^ and Russia^51^ production quantity for 2018 was retrieved. For India the ethylene market volume size for 2023 was available (https://www.chemanalyst.com/industry-report/india-ethylene-market-92). Further comprehensive worldwide dataset was found for *propylene* capacities from propane dehydrogenisation plants from 2021^52^. However, this technology was responsible for only 5% of the global propylene production in 2014^53^. Thus, this propylene capacity was extended based on additional sources where possible. Additional sources were found for Indonesia and Japan^49^, European countries^14,54^ (https://www.hip-petrohemija.com/tehnologija/proizvodna-linija/etilen.22.html, https://www.icis.com/explore/resources/news/2021/03/24/10621155/italy-s-petchems-units-face-uncertain-future-as-porto-marghera-set-to-close/), India^55^, Russia^56^, China (https://www.icis.com/asian-chemical-connections/2023/03/global-oversupply-of-petrochemicals-to-hit-218m-tonnes-in-2023-the-highest-in-any-year-since-1990/), and USA^57^. Assuming that global production of ethylene and propylene is equivalent to their global demand (of around 270 million tonnes in 2018^58,59^) with a 100% usage rate of the elaborated installed capacities the sum of obtained national production quantities accounts for 81% of the global ethylene and propylene production in 2018.

*Aromatics* production capacities include benzene, toluene, ortho-, para- and meta-xylenes hydrocarbons as main products^60^. No publicly available database or world-wide summary of the national production capacities or quantities was found. For European countries national production capacities were constituted from multiple sources^54,61,62^ (https://corporate.exxonmobil.com/news/news-releases/2017/0827_exxonmobil-completes-acquisition-of-one-of-the-worlds-largest-aromatics-plants, https://www.cepsa.com/en/press/Cepsa-starts-up-second-up-metaxylene-production-unit-at-San-Roque). Other used references are statista presentations of production quantities in 2018 for Korea^63,64^, USA^65–67^, reports for Japan^68^, Russia^69^, India (https://www.ceicdata.com/en/india/petrochemical-production-by-product/petrochemical-production-aromatics-mixed-xylene, https://www.ceicdata.com/en/india/petrochemical-production-by-product/petrochemical-production-aromatics-toluene, https://www.chemanalyst.com/industry-report/india-paraxylene-market-51, https://www.icis.com/asian-chemical-connections/2011/11/india-benzene-exports-set-to-r/) as well as other references for a mix of other countries^70^ (https://www.icis.com/asian-chemical-connections/2023/03/global-oversupply-of-petrochemicals-to-hit-218m-tonnes-in-2023-the-highest-in-any-year-since-1990/, https://corporate.exxonmobil.com/news/news-releases/2017/0827_exxonmobil-completes-acquisition-of-one-of-the-worlds-largest-aromatics-plants, https://icis.shorthandstories.com/2023-global-market-outlook-aromatics). It shall be underlined that available data were a mixture of information regarding production quantities and capacities, for year 2018 but also some more recent or older years. Furthermore, some of the reports are covering only newly built capacities or certain companies. Assuming a maximal usage rate of the installed capacities, a coverage rate of 72% of the global aromatics production capacity presented by statista^71–73^ is achieved.

To capture the full energy demand of the chemical and petrochemical subsector, “*other chemicals*” are included based on an allocation in which the above elaborated five chemicals account for 70% and other chemicals for 30% of the total.

Summarizing the data collection regarding national production quantities per process route, the certainty of the collected and elaborated values for 2018 as well as the geographical resolution of the original data from the utilized data sources is presented in Table 2.

**Table 2:** Certainty of the national production quantity values as assessed by the authors.

Branch	Products	Certainty of the national production quantity values	Original geographical resolution
Iron and steel	Primary steel	High	National
	Secondary steel	High	National
Aluminium	Primary aluminium	High	National
	Secondary aluminium	Low	Site, national, global
Copper	Primary copper matte Primary refined copper Secondary copper matte Secondary refined copper	High High High High	National National National National
Cement	Cement	High	Site, national, global
Glass	Container glass	Low	Global
	Flat glass	Low	Global
Lime	Lime	High	National
Pulp	Chemical pulp	High	National
	Mechanical pulp	High	National
	Recycled pulp	High	National
Paper	Graphical paper	High	National
	Hygiene paper	High	National
	Packaging paper	High	National
	Technical packaging paper	High	National
Chlorine	Chlorine	High	National
Methanol	Methanol	Low	Regional
Ammonia	Ammonia	High	National
Olefins	Ethylene Propylene	Mid	Site, national, global Site, national, global
		Low	
Aromatics	Benzene Toluene(o-, p-, m-) xylene	Low Low Low	Site, national, global Site, national, global Site, national, global

### Step 2: Specific energy demands for BAT and theoretical minimum

In this step BAT process routes for each of the considered industrial products are elaborated. Initially, specific energy demands (including electricity, fuel or thermal energy and feedstock demand) of the current BAT process routes are estimated. Each of the BAT process routes is supplemented with information on the theoretical minimum specific energy demand, primarily expressed in terms of thermal energy and feedstock. Following subsections briefly describe how specific energy demands are derived and which data sources are utilized. Subsequently, the share of required fuels (coal, gas, oil etc.) in total BAT fuel or thermal energy demand is determined. Because some processes can employ different fuels for heat production (for example, smelting), the actual selection of fuel depends strongly on its regional availability and price. To capture this flexibility, maximum fuel shares are defined for these cases. These values enable energy system modelers to explore potential fuel switching while maintaining consistency with observed practices. For these cases, differences in energy requirements among different fuels, which arise from variations in combustion or conversion efficiency, are neglected because they are rather small and not well documented in the literature. Feedstock demands of the production routes are considered only if the energy for their production is considered as a part of the production route chain. Process routes utilized in the calculations are shown in the reference energy system diagrams.

#### Iron and steel

**Fig. 4:**
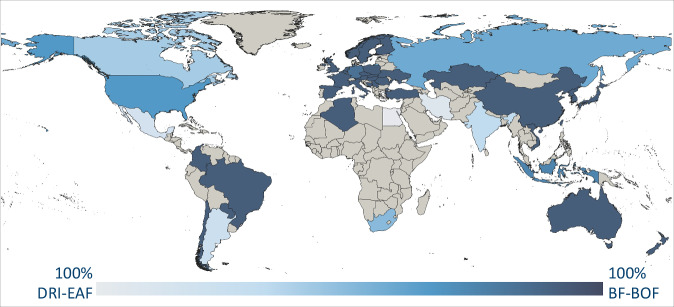
Steel process route shares 2018. DRI-EAF – Direct Reduction Iron – Electric Arc Furnace, BF-BOF – Blast Furnace – Basic Oxygen Furnace. Grey stands for absence of steel production by any route. Data source:^21^.

For the primary steel production, we consider the two most relevant process routes^74^: (a) iron production by blast furnace (BF) followed by steel production in basic oxygen furnace (BOF) and (b) iron production by direct reduction (DR) followed by steel production in electric arc furnace (EAF). It is assumed that the national share between these two routes is equivalent to the share between the PI and DRI production of the corresponding country^21^. Figure 4 shows the resulting global process route shares for the steel industry.

The scope of our energy demand estimation considers the process steps from iron ore to the crude steel but excludes the mining processes. Sintering and pelletizing would not necessarily be included by this definition. However, we consider energy requirements of both processes, as the energy demand of both iron ore preparing processes differ between the routes^75^.

**Fig. 5:**
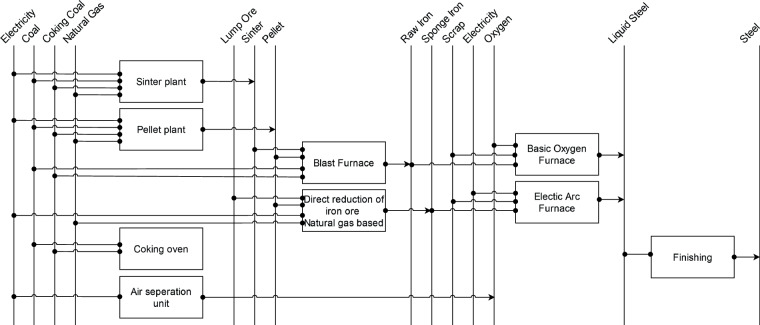
Reference energy system for iron and steel production.

For the sinter, average pellet and coke demands from Best available techniques (BAT) reference document for iron and steel production^20^ [t_educt/t_steel] are multiplied with the specific energy demands [GJ/t_educt] given in Moya *et al*.^76^. Energy demands for the other processes, including the iron and steel making itself, are compiled based on BAT reference document for iron and steel production and Otto *et al*.^20,77^. Graphical representation of the reference energy system for iron and steel production is given in Fig. 5. Theoretical minimal specific energy demand is sourced from Fruehan *et al*.^78^.

#### Non-ferrous metals

**Fig. 6:**
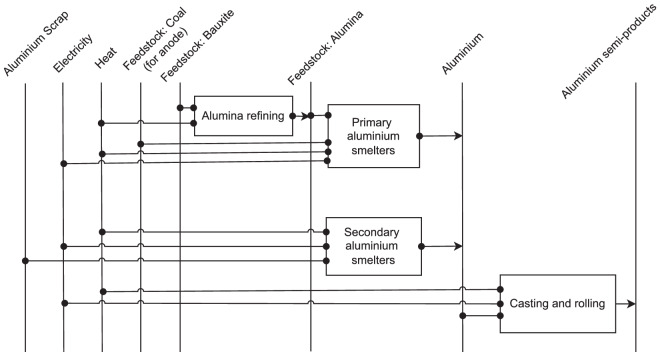
Reference energy system for aluminium production.

**Fig. 7:**
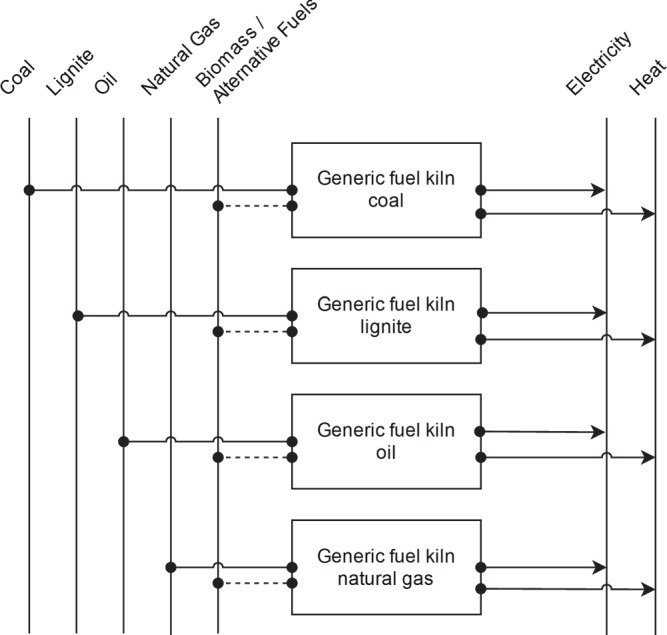
Reference energy system for heat production.

Production of the primary *aluminium* starts with mining of bauxite ore; however, as part of mining activities, this energy demand lies outside of the system boundaries of this study. In the Bayer-process bauxite ore is milled, digested, filtrated and subsequently undergoes precipitation and calcination processes to produce alumina^25^. Typically, primary aluminium is manufactured from alumina by the aluminium electrolysis by Hall-Héroult method^25^. Specific energy demand for alumina refining is sourced from BAT Reference document^79^; for electrolysis and aluminium casting and processing it is based on Kuder’s BAT descriptions^80^. Due to the lack of other information, the latter values were split into electricity and fuel demand based on German average energy demands for the respective processes^25^. Reference energy system for aluminium production is given in Fig. 6. Since heat is input used in many production processes, this step of energy conversion is separately shown in Fig. 7. Theoretical minimal specific energy demand for the Hall-Héroult process includes both electrolytic work as well as thermal energy, whereas other processes include only the thermal energy^81^.

**Fig. 8:**
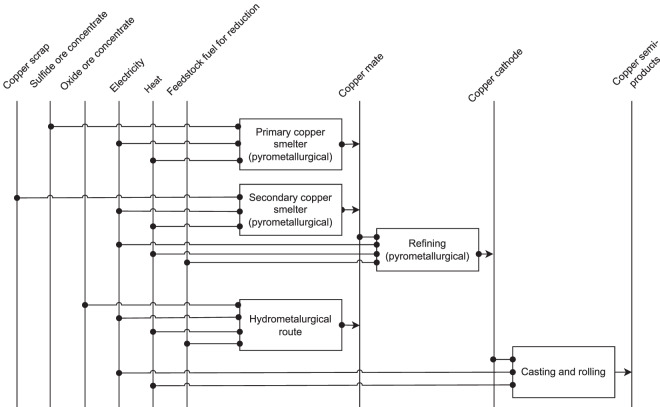
Reference energy system for copper production.

The type of utilized copper ore determines the copper production route – pyrometallurgical route uses sulphide ore whereas hydrometallurgical route utilizes oxide ore, see Fig. 8. Main steps of the pyrometallurgical route include concentration of the ore by comminution and flotation, smelting, converting, fire refining and finally electrorefining^28^. The hydrometallurgical route includes concentration of the ore by leaching, then solvent extraction and finally electrowinning^28^. This study does not consider comminution processes, since based on weir^82^ they belong to the mining subsector. Specific energy demand of the copper product depends on the production processes the metal undergoes. Since some manufacturers produce copper which goes only through smelting, we have to diversify the specific energy demands of the production processes. However, BAT specific energy demands found for primary and secondary copper production were not split between the production processes and were for the pyrometallurgical route only. Moreover, they were total energy demands and did not state the share of electricity and fuel used. Therefore, following considerations were made to access BAT specific energy demands needed for an accurate energy demand estimation.

Specific total energy demands for the BAT for pyrometallurgical primary and secondary copper productions are based on Kuder^80^. These values are split on groups - smelting processes and refining processes, based on average specific energy demands from Moreno-Leiva *et al*.’s paper^28^. Distribution of the energy demand to electricity and fuel demand is for the refining processes based on Moreno-Leiva *et al*.’s work^28^. In lack of more general data and since German copper industry applies pyrometallurgical route, the electricity to fuel ratio of the pyrometallurgical smelting processes is based on the corresponding German values^25^.

For hydrometallurgical route the same efficiency enhancement between average and BAT specific energy demand is assumed to be applicable as for the pyrometallurgical route. Thus, BAT specific energy demand of the hydrometallurgical route is obtained by scaling its average value from Moreno-Leiva *et al*.’s paper^28^ by the ratio between Kuder’s BAT and Moreno-Leiva *et al*.’s average specific energy demand for pyrometallurgical copper production. Distribution of this energy demand to electricity and fuel demand is also based on Moreno-Leiva *et al*.’s work^28^.

Based on German reports, specific energy demand for casting and processing is similar within the non-ferrous metals’ branches^25^. Thus, total BAT specific energy demand for casting and processing of aluminium from Kuder’s work^80^ is assumed to apply for copper, as no copper specific value was found. The electricity to fuel ratio is based on the German distribution of the energy demand for non-ferrous metals casting and processing^25^.

Theoretical minimal specific energy demand for primary copper smelters is sourced from Alvarado *et al*.^83^, while for secondary copper smelting, for refining and foundry casting it is estimated based on Schifo *et al*.^84^. Theoretical minimal thermal energy for leaching, solvent extraction and electrowinning is based on Beukes and Badenhorst^85^.

#### Non-metallic minerals

**Fig. 9:**
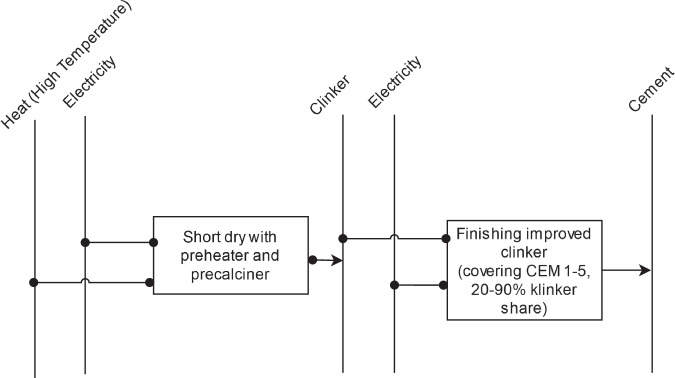
Reference energy system for cement production.

*Cement* production involves two primary stages: clinker production and cement manufacturing. The intermediate product, clinker, can be produced using either wet or dry processes with limestone and clay as material inputs. Dry processes are further categorized into long dry, Legpol, short dry, and short dry with preheater and precalciner methods. Each of these methods has varying levels of electricity and fuel demand, with the wet process demanding the most energy, leading to its discontinuation in many countries. Cement is then produced through a finishing process using clinker as the material input, such as in the finishing Portland process. Currently, the short dry process with preheater and precalciner is the most efficient^86^ and is thus considered the BAT. A reference energy system for cement production as considered in this study is shown in Fig. 9. Theoretical minimal specific energy demand for the clinker production is sourced from the European Cement Research Academy report^87^, while for the cement it is sourced from Worrell *et al*.^88^.

**Fig. 10:**
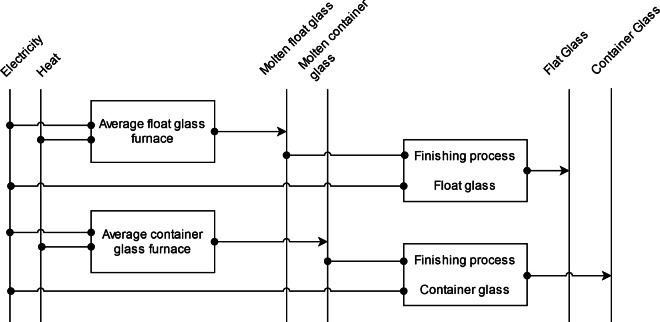
Reference energy system for glass production.

The specific energy requirements of flat *glass* are almost twice as high as those of container glass^31^, while global flat glass production capacities are approximately 1.2 times those of container glass production capacities^89^. The routes differ primarily in the configuration of the furnaces, the fuel, the trajectory of the glass, and the heat recovery techniques employed^31^. However, as previous examination of the available glass production levels or capacities has revealed, it was not possible to differentiate between the two routes for the national distribution of the global glass production. Consequently, based on the global share of routes^89^, a weighted average specific energy demand between flat and container glass, based on Zier *et al*.^21^, is assumed, see Fig. 10. Theoretical minimal specific energy demand of glass production is sourced from Baron *et al*.^90^.

**Fig. 11:**
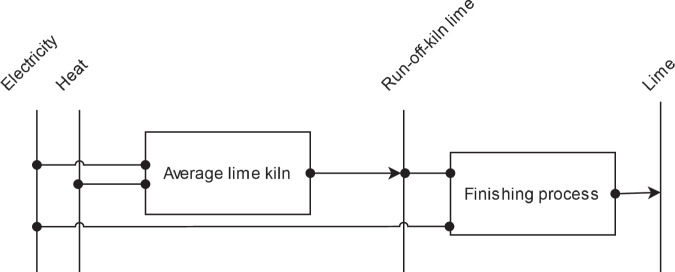
Reference energy system for lime production.

The global *lime* industry uses several different kiln types for the same operation: Reducing limestone to lime. The two main different process characteristics are the vertical and the horizontal arrangement of the kiln, while the detailed process route is dominantly determined by the exact lime composition to be achieved. For Europe, for example, the *Parallel flow regenerative kiln* is the most used route^91^. Lacking exact data on TIAM regional kiln technologies distribution on a global level and facing the relatively large range between the specific energy demands of the process routes, an average energy demand of available kilns from Stork *et al*.^91^ is chosen as the BAT value. A reference energy system for lime production is given in Fig. 11. Theoretical minimal specific energy demand is obtained from the same reference^91^.

#### Pulp & Paper

**Fig. 12:**
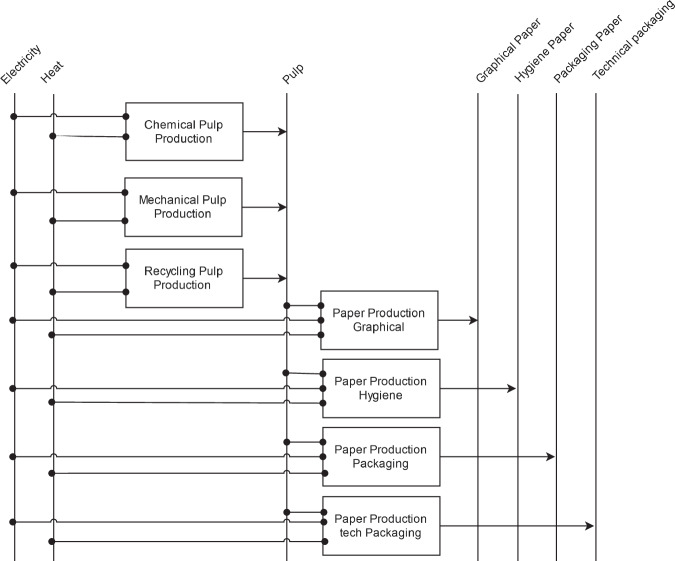
Reference energy system for pulp and paper production.

For each grade of pulp and paper, see Fig. 12, the specific electricity and heat demand of the respective BAT is used, as presented by IEA^92^. Although the theoretical minimal specific energy demand varies among pulp grades, paper is assigned a single value^93^. It is worth noting that for recycled pulp, no theoretical minimal value is defined, as there is neither bond-breaking nor chemical reaction involved.

#### Chemicals

**Fig. 13:**
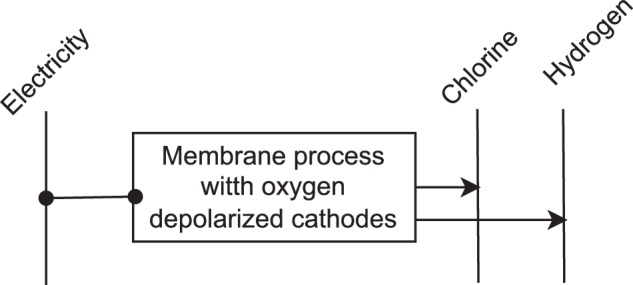
Reference energy system for chlorine production.

*Chlorine* is primarily produced using chlor-alkali electrolysis process operated solely by electricity without fuels usage, see Fig. 13. Brine solution is used as a feedstock producing chlorine, hydrogen and sodium hydroxide.

There are four different types of the electrolysis processes for chlorine production  1. Among these processes, membrane process with oxygen depolarized cathodes is the most energy efficient and considered as BAT. The specific energy demand for the BAT has been taken from Bazzanella *et al*.^5^. Theoretical minimal specific energy demand of the chlorine production equals the thermodynamic minimum of the electrolysis^94^.

**Fig. 14:**
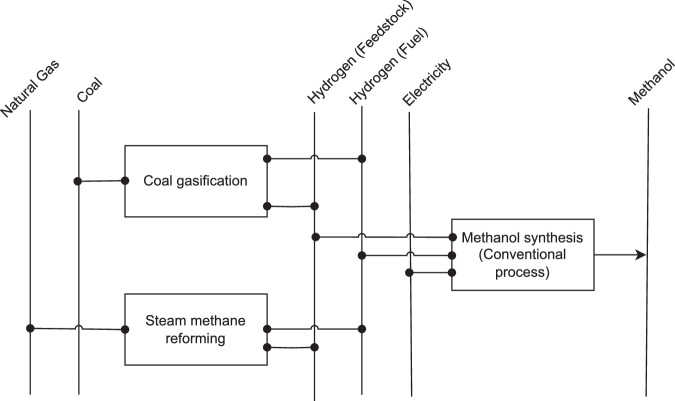
Reference energy system for methanol production.

*Methanol* is primarily produced through methanol synthesis. The traditional method uses hydrogen and CO_2_ along with electricity, where hydrogen is generated via Steam Methane Reforming (SMR) or coal gasification. In SMR, natural gas serves as the feedstock, while coal is utilized in the gasification process, see Fig. 14. This conventional method results in methanol with a high CO_2_ footprint due to the hydrogen feedstock.

New technologies, such as Power-to-Methanol and Biomass-to-Methanol, offer greener alternatives. In Power-to-Methanol, hydrogen is produced by electrolysis using green electricity. In Biomass-to-Methanol, biomass energy is used as feedstock to generate green methanol. Despite these advancements, as of 2018, methanol production relies entirely on the conventional synthesis process. Outside of China, hydrogen for methanol production is sourced from the SMR process, whereas China leverages its coal resources for hydrogen production through gasification.

For the BAT selection, the conventional methanol synthesis process is considered. Due to data availability, European averages for the specific energy demand of coal, natural gas, and electricity are used as the BAT benchmark^95,96^. In terms of fuel usage, coal is exclusively used in China for methanol production, while natural gas is used in the rest of the world. Theoretical minimal thermal energy demand of methanol production is set to zero as the process is exothermic and theoretically no energy is needed. Feedstock demand is assumed to equal the BAT value.

**Fig. 15:**
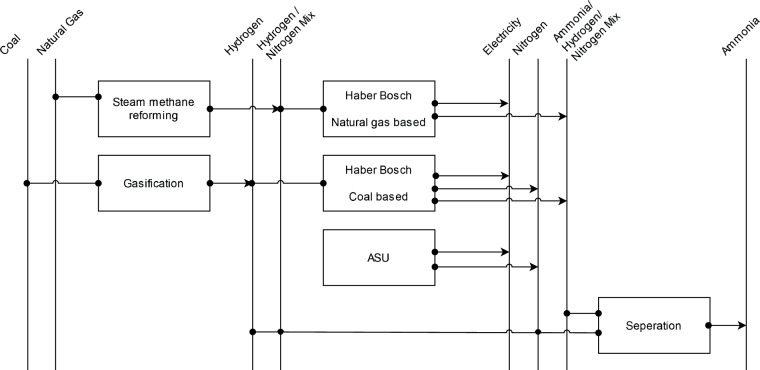
Reference energy system for ammonia production.

Thanks to the Haber-Bosch-process the basic process of producing *ammonia* is very standardized globally. The process includes the provision of hydrogen and nitrogen, which subsequently combine to form ammonia within the Haber-Bosch reactor. The biggest differences between regions are emerging due to a different source for hydrogen as it is the case for methanol. Especially China uses coal gasification to produce hydrogen, while the rest of the world mainly uses the natural gas-based methane-steam reduction route to provide hydrogen^97^. Following this, the energy requirements from IEA^97^ for the coal-based route are assumed for China, while the energy requirements for the gas-based route from the same source are assumed for the rest of the world, as represented in Fig. 15. Total theoretical minimal specific energy demand of the Haber-Bosch-process is sourced from Rouwenhorst *et al*.^98^.

**Fig. 16:**
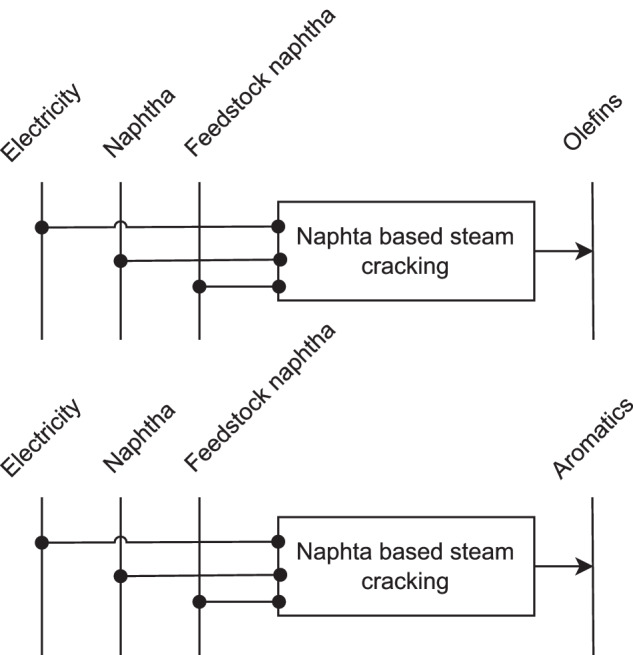
Reference energy system for olefins and aromatics production.

Both *olefins* and aromatics, together termed as high value chemicals (HVC) can be sourced in multi- or single-product processes, or as by-products of the refinery sector^6^. Ethylene is produced almost exclusively from steam crackers^6^. Propylene originates 50% from steam crackers and 39% from fluid/deep catalytic cracking as a by-product of refining operations^53^. Single-product routes propane dehydrogenation and olefin metathesis make additional 5% each^53^. Olefins can also be produced by methanol-to-olefins process, which is due to the abundant access to cheap coal for methanol production, done only in China^6^. Chemical feedstock is used to physically constitute chemical products composed of carbon and hydrogen. Its choice depends on one hand on the availability and costs of the fuels. On the other hand, the feedstock choice affects the total production yield and, in case of multi-product processes, the diversity of the obtained products^6^. Single-product processes can consume relatively low amounts of feedstock per unit of product, especially when feedstock and the product have a similar chemical structure^6^. To determine the BAT for olefine production, the lowest BAT value of the technologies and feedstocks most widely applied in 2018 was selected. Steam cracking based on naphtha showed lower BAT specific energy demand in comparison to the ethane-based process^99,100^. Methanol-to-olefins process contributed to less than 5% of olefine production in China 2018^101,102^. Moreover, its specific energy demand was higher than for the naphtha steam cracking, based on Dechema’s studies^5,95^. Thus, naphtha steam cracking was considered to be the BAT. Its specific energy demand was adopted from Dechema’s study^5^. The reference energy system for olefine production is represented in the upper part of the Fig. 16. BAT electricity demand is assumed to be 1 GJ per ton of product^95,99^; the rest is considered to be fuel. Feedstock demand relies on the Dechema’s study^5^. Theoretical minimal specific thermal energy demand is sourced from Bolson *et al*.^103^, while the feedstock demand is assumed to be equal as of the BAT.

*Aromatics* are mostly produced by fluid catalytic cracking and continuous catalytic reforming in refineries^6^. However, more than 70% of the aromatics produced by catalytic reforming are utilized for boosting the gasoline’s octane value and thus go to the gasoline pool^60^. Naphtha steam cracking is the main petrochemical source for aromatics^53^. Naphtha catalytic cracking (NCC), which makes usage of catalyst, requires 15% less energy than the world’s BAT naphtha steam cracker, as well as almost 25% less naphtha feedstock per ton of HVC produced^6^. Yet, the process is relatively new and complex and only one such commercial plant is operating in South Korea^6,104^. Furthermore, while NCC does produce some aromatics, it primarily converts heavy hydrocarbon fractions into lighter products. Thus, this process was left from the BAT consideration for 2018. Total BAT specific energy demand of naphtha steam cracking is taken from Dechema^5^. The reference energy system aligns with the one for olefines production, see Fig. 16. Based on aromatics extraction process by Saygin *et al*.^4^, the electricity is assumed to constitute approximately 5% of this energy. Feedstock demand per ton of aromatics is, accordingly to the same study^4^, assumed to be the same as per ton of olefins. Theoretical minimal specific thermal energy demand is set to zero, since benzene and mixed xylenes have negative and paraxylene a low positive theoretical minimum value^105^. The theoretical minimal feedstock demand is assumed to be equal to that of the BAT.

### Step 3: Resulting national energy demands and regional inefficiency factors dataset

Once the production quantities as well as BAT and theoretical minimum specific energy demand values are determined, the national BAT and theoretical minimum energy demand for each production route is calculated by their multiplication, respectively (see Fig. 1). The calculated total fuel demand for BAT is allocated to specific energy carriers on a regional basis. Where multiple energy carriers can be used for the same process, the allocation follows the regional and subsectoral energy-carrier shares reported in the IEA energy balances^13^ for the year 2018. This ensures that the estimated fuel mix reflects the prevailing energy context of each region. Specifically, the methanol and ammonia coal-based production routes are attributed to the Chinese industry, whereas the natural gas-based routes are attributed to the rest of the regions. Theoretical minimum energy demand is primarily expressed in terms of thermal energy and feedstock, with no distinction of the possible energy carriers. Next, the inefficiency factors are assessed as follows.

**Fig. 17:**
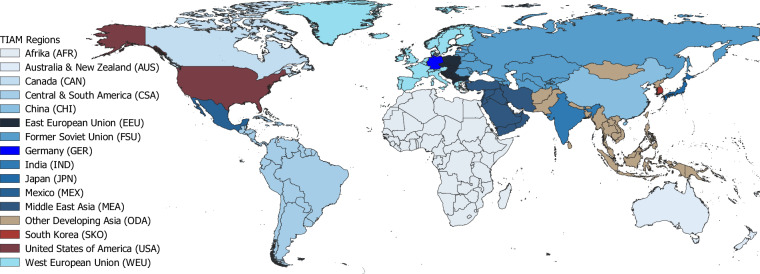
TIAM regions. TIAM regions are used for geographical aggregation of the energy demands and consumptions as well as inefficiency factors.

The inefficiency factor in our work represents the ratio between statistical energy consumption and the calculated energy demand based on the BAT or the theoretical minimum. IEA energy balances^13^ are available only for the industrial subsectors. Therefore, the calculated energy demands of the branches are aggregated per industrial subsector, accordingly to the Table 1, and summed up to correspond to the statistical energy consumption. Moreover, for BAT calculations, self-generated electricity in industrial sites can distort the distribution of energy consumption between electrical energy and fuel in the energy statistics. To avoid this issue, total energy consumption and total energy demand are compared. Nevertheless, for clarity and completeness, we also estimate inefficiency factors separately for electrical energy and for fuel use. Additionally, data are geographically aggregated in regions, as represented in the Fig. 17. The proposed method would be applicable also on the national basis, resulting in national inefficiency factors. However, the necessity for the regional aggregation lies in the underlying data availability. On one hand, the national production quantities estimation certainty, as summarized in Table 2, varies across the industrial subsectors. On the other hand, the utilized IEA Energy balance^13^ statistics is proprietary, which is why we demonstrate the method using the aggregated data.

The ratio between the statistical energy consumption ${IEA\_EB}$ and the calculated energy demand (based on BAT or theoretical minimum) ${CED\_}\min$ gives the inefficiency of the subsector, see Eq. (1) upper case line. ${Regions}$ stands for considered TIAM regions, and ${Ineff}$ for the inefficiency factor. The average statistical specific energy consumption should be greater than the specific energy demand of the BAT or the theoretical minimum, meaning that inefficiency factor should always be greater than 1. Yet for some regions and subsectors the calculated inefficiency factor falls below 1. One possible explanation is that the industrial plant stock in these countries is more efficient than the BAT used as a benchmark in this study. The specific energy demands of the BAT production processes referenced in this work are based on literature assumptions rather than on the thermodynamic minimums. Even so, the reason of this discrepancy is more likely related to the quality of the reporting. This is further supported by the fact that some of the calculated inefficiencies, even when based on theoretical minimums, are also below 1. Countries should report energy consumption by industrial subsector; nonetheless, the IEA energy statistics also include a catch-all category for energy consumption - Not Elsewhere Specified (NES). The share of NES energy consumption should be as low as possible. However, as discussed in the Technical Validation section, for some of the countries this value makes more than 50% of the total statistical industrial energy consumption. Therefore, if the calculated inefficiency factor is below 1, statistical information about the subsector in the region was considered incomplete and adjusted inefficiency factor was set to be average of the other inefficiency factors being greater equal 1, see Eq. (1) lower case line. For completeness, the elaborated dataset also includes the raw inefficiency factors. The IEA already discussed for the chemical and petrochemical industrial subsector the inconsistencies in international energy statistics as well as other factors which possibly lead to energy demand based on BAT being above the statistical consumption^4^.

1 $${\rm{\forall }}\,r\,\in \,Regions:{Ineff}_{r}=\{\begin{array}{cc}\frac{IEA{\rm{\_}}E{B}_{r}}{CED{\rm{\_}}{min}_{r}} & if\,\frac{IEA{\rm{\_}}E{B}_{r}}{CED{\rm{\_}}{min}_{r}}\ge 1\\ \frac{{\sum }_{i=1}^{n}{Ineff}_{i}}{n},i\,\in \,Regions|\frac{IEA{\rm{\_}}E{B}_{i}}{CED{\rm{\_}}{min}_{i}}\ge 1 & if\,\frac{IEA{\rm{\_}}E{B}_{r}}{CED{\rm{\_}}{min}_{r}} < 1\end{array}$$

**Fig. 18:**
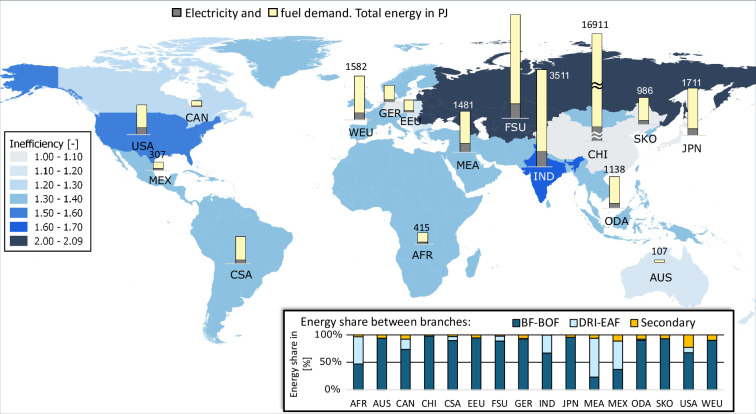
Calculated final energy demand and BAT inefficiency factors for iron and steel subsector.

**Fig. 19:**
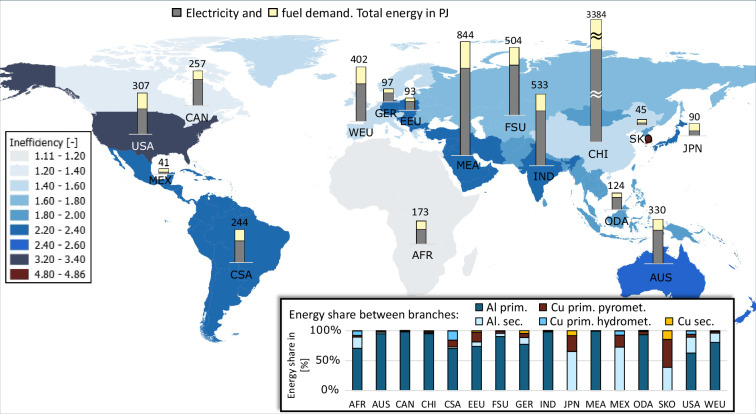
Calculated final energy demand and BAT inefficiency factors for non-ferrous metals subsector.

**Fig. 20:**
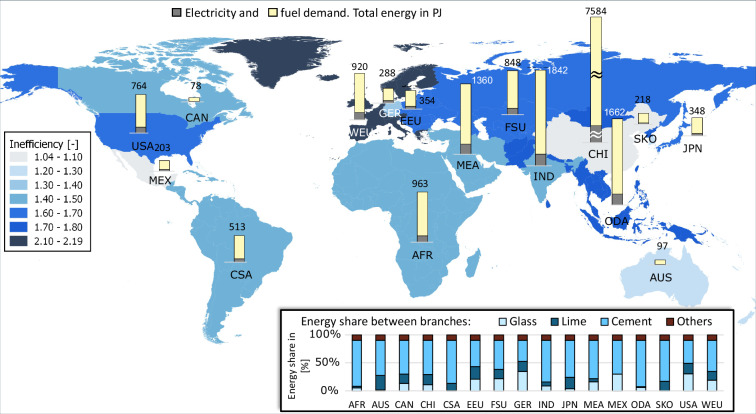
Calculated final energy demand and BAT inefficiency factors for non-metallic minerals subsector.

**Fig. 21:**
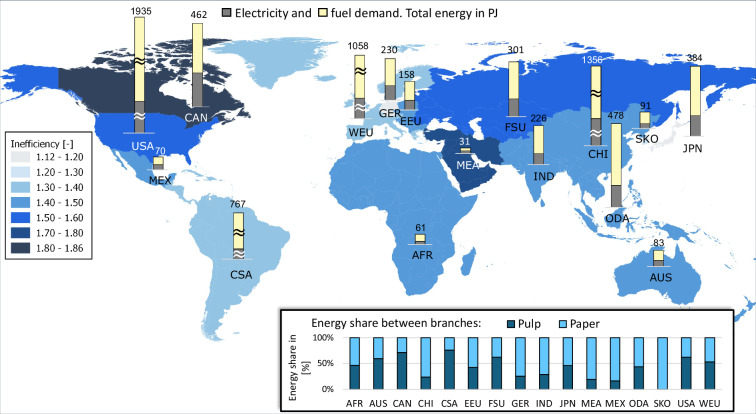
Calculated final energy demand and BAT inefficiency factors for pulp and paper subsector.

**Fig. 22:**
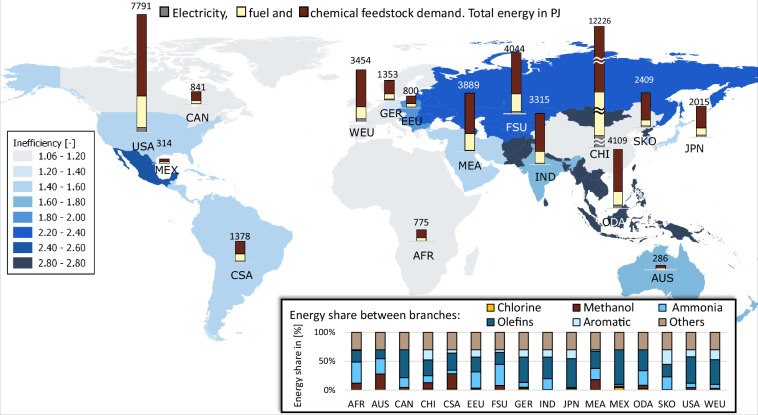
Calculated final energy demand and BAT inefficiency factors for chemical and petrochemical subsector.

Ultimately, calculated energy demands based on BAT are multiplied by the BAT (total) inefficiency factors to obtain final (scaled) energy demands for each industrial subsector and each TIAM region. Figures 18–22 depict globally the final energy demands of the industrial subsectors. In the background the TIAM regions are shaded based on the estimated BAT total inefficiency factor of the subsector. Bar charts on top of the regions in the map depict calculated electricity and fuel demands. The bar chart in the right bottom of the figures illustrates the distribution of the total final energy demand on different industrial branches of the subsector. For some of the regions, typically China, the energy demand was substantially higher than for the others. Therefore, the maximal axes value for bar charts marked with “≈” sign, was selected to be higher than for the others.

For the iron and steel subsector on Fig. 18 it is notable that the eastern regions China, Former Soviet Union and India show highest level of final energy demand based on the corresponding production activity. Fuel usage is by far higher than the electricity consumption. While China, South Korea, Japan and Germany exhibit the highest energy efficiency (lowest inefficiency factors), the Former Soviet Union shows the highest inefficiency factor. Production route BF → BOF is generally most widespread, although DRI → EAF dominates the steel production in the Middle East Asia (MEA), Mexico and Africa. Secondary steel production makes rather a very low process route share, except for the USA.

The non-ferrous metals subsector reveals, see Fig. 19, untypically for the other subsectors, notably higher electricity than fuel demand. Furthermore, it states very high inefficiency factors. This comes on the first place from the assumption that aluminium and copper industry alone are responsible for the total non-ferrous metals energy demand. The variety of non-ferrous metals, the diversity of their geographical distributions as well as different specific energy demands for their production made a global assumption regarding the share which aluminium and copper industry have in this subsector very complex. On one hand, this result indicates the necessity of cautiousness when interpreting inefficiency factors. On the other hand, high inefficiency factors can have other causes too. Clear distinction between statistically reported energy used for mining and for non-ferrous metals processing could be disputable, because some sites include parts of both process chains. Moreover, although basic metal processing after the refining is assumed, the additional post-processing into aluminium or copper items is not considered. Statistical reporting with global coverage of such items is missing as well as assessment of specific energy demands per product unit would require specialisation in too many distinct application fields. This is especially observable on the example of South Korea, which has the highest calculated inefficiency factor. South Korea has no primary aluminium production^16^, but it has considerable aluminium plates production^106^, which is due to the global approach and available databanks not considered in this study. Summarising, although it encompasses some uncertainties, this approach allows us to represent the total energy demand of an industrial subsector, as intended, by implementing and analysing only its few main branches.

As further seen from Fig. 19 most of the energy for global non-ferrous metals industrial subsector is required for the primary aluminium production. However, Japan, Mexico and South Korea produce only secondary aluminium as well as copper. Although hydrometallurgical copper production route has a subordinate global energy demand, it plays an important role in Africa and Central & South American (CSA) region.

Among the highest energy consumers in non-metallic minerals sector, see Fig. 20, alongside the already mentioned China, India and the MEA, is Other Developing Asia (ODA). Africa takes part in the energy demand to a higher extent than in the previous subsectors. Fuel usage is notably more dominant than the electricity demand. Cement production takes place in all regions. In this subsector, China, South Korea and Mexico are the most efficient producers (having the lowest inefficiency factors), whereas West Europe has the highest inefficiency factor.

The energy demand for pulp and paper production, see Fig. 21, is more evenly distributed. The share of electricity and fuel in the total energy demand is more balanced than in the other subsectors. Japan has the highest energy efficiency (lowest inefficiency factor), while the highest estimated inefficiency factor is attributed to Canada. South Korea produces solely paper whereas other regions produce both pulp and paper products.

The chemical subsector, see Fig. 22, has very high total final energy demand. The usage of the chemical feedstock is approximately twice as high as electricity and fuel usage together. Highest inefficiency factor is estimated for the ODA region. Production of olefins holds the highest share in the final energy demand (above 30%). Aside from the “other chemicals”, the remaining final energy demand is distributed among ammonia, aromatics, methanol and finally chlorine production in descending order. Whereas olefine production is existing in every region and ammonia significantly contributes in almost all of them, the rest of the elaborated chemicals are differently represented across the regions.

**Fig. 23:**
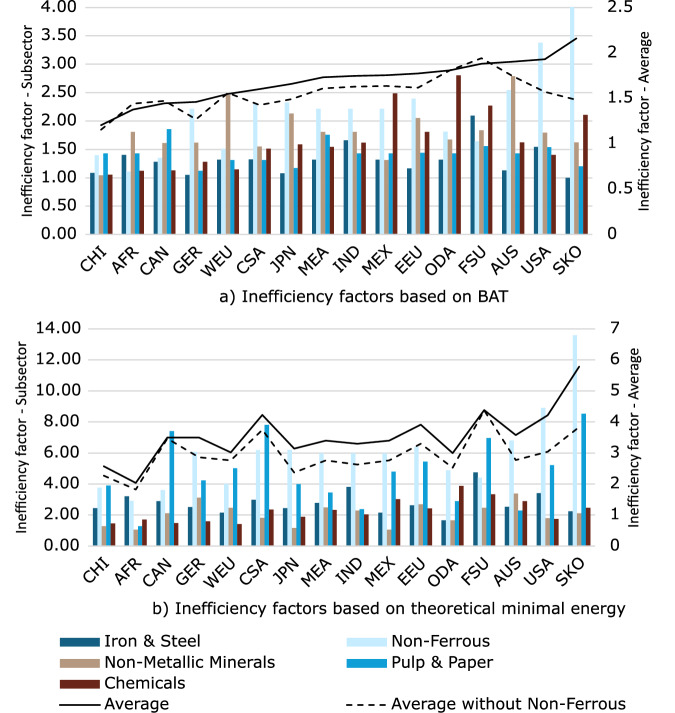
Cross-sector overview of inefficiency factors. Inefficiency factors based on a) BAT, b) theoretical minimal energy.

Figure 23 provides a summary of inefficiency factors across regions and sectors, based both on a) BAT and b) theoretical minimal energy. Regions are sorted based on BAT based average inefficincy factors, which are discussed first. The South Korea has the overall highest inefficiency factors, while China demonstrates the lowest inefficiency factors. Significant variation exists between sectors within the same region—for example, Australia and New Zealand (AUS) has a low inefficiency factor in the iron and steel subsector, while it has a notably high inefficiency factor in non-metallic minerals subsector. The non-ferrous subsector generally shows particularly high inefficiency factors. Average inefficiency factors range from 1.20 to 2.16; however, when the non-ferrous sector is excluded, the range narrows to between 1.15 and 1.94. As expected, the inefficiency factors based on the theoretical minimum are significantly higher than those based on BAT. The regional averages range from 2.04 to 5.79, decreasing to 1.82 to 4.38 when the non-ferrous subsector is excluded. The pulp and paper subsector stands out as the second highest in terms of inefficiency factors calculated based on theoretical minimal energy. It is important to highlight that the diversity of the manufacturing industry in certain regions can result in high inefficiency factors, which compensate for industrial branches that are not explicitly modelled.

## Data Records

The data produced by this study are stored on Zenodo under 10.5281/zenodo.15524913^107^. Users can download the repository and use the data on their local system. The repository structure follows the study flow. There are two main folders as given in Table 3. Additionally, territories considered under the aggregated TIAM regions are given in file TIAM_regions.csv.

**Table 3:** Structure of the data records.

Folder	Data description
01_Production_quantities	Twelve files, grouped by industrial subsector, encompassing national production quantities per industrial branch and product.
02_Energy_calculation	The first file includes specific energy demands for the production of the above-mentioned industrial products by the BATs as well as for the theoretical minima. The second file consists of regional industrial energy demands per branch and production process, grouped by industrial subsector, and calculated based on the BATs and the theoretical minima. Distinguished are, for BAT energy demand, electricity and different main fuel types (for energy use as well as for chemical feedstock). The third file includes inefficiency factors (raw and adjusted) per TIAM region and per industrial subsector, for BAT and for the theoretical minimum. It also contains energy demands calculated for the BAT and for the theoretical minimum as well as final (recalculated) energy demands, per TIAM region, industrial subsector, branch and process route, for electricity and total fuel including feedstock. The fourth file includes validation calculations.

## Technical Validation

**Fig. 24:**
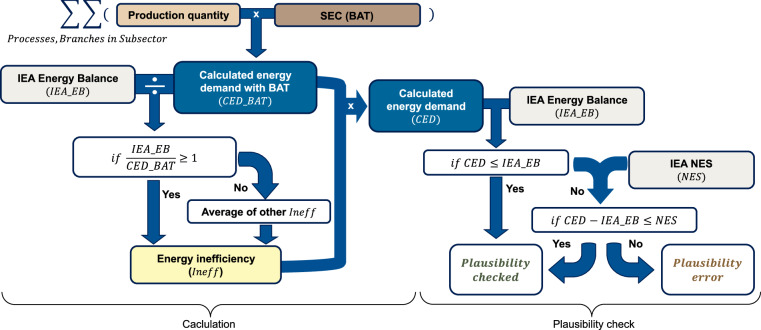
Calculation process and plausibility check.

To ensure the plausibility of our calculations, for countries with a higher calculated energy demands than the IEA energy statistics, we initially verified if there are national reports regarding energy demand of the respective industrial subsectors. Some of the examples follow. The calculated India’s non-ferrous metals total energy demand based on BAT accounts for 205 PJ. According to the IEA energy statistics, this energy consumption was around 70 PJ^13^. However, ten biggest aluminium producing energy consumers in India consumed already roughly 320 PJ in 2007^108^. In 2017 Hindalco Industries, Indian aluminium and copper producer, was alone responsible for an energy consumption of around 270 PJ^109^, thus highly exceeding the IEA energy statistics of the subsector in India’s energy statistics. Similarly, the calculated Middle East Asia’s non-ferrous metals total energy demand with BATs accounts for around 325 PJ, whereas IEA statistics sum to around 80 PJ. However, EGA Emirates global aluminium company in United Arab Emirates alone consumed in the period of 2018–2021 around 130 PJ per year^110^. The reports thus confirmed higher energy consumption of the affected industrial subsectors in comparison to the IEA statistics. Hence, our approach to consider our calculations as more accurate and to scale them up by an inefficiency factor above 1 proved reasonable. The left side of Fig. 24 summarizes the calculation process ending with Calculated Energy Demand (CED).

**Fig. 25:**
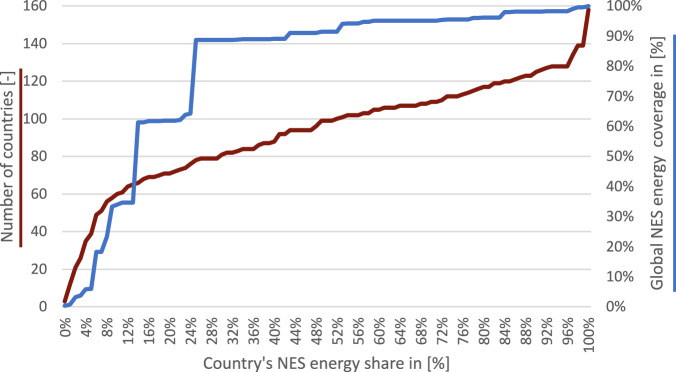
NES energy demand shares in the total industrial energy demand. The diagram shows cumulative number of countries (red) and cumulative global NES energy demand coverage (blue), when per country NES energy demands are sorted by the NES energy share in the total industrial energy consumption.

Subsequently, we checked if the total final calculated energy demand exceeds the industrial energy balance provided by the IEA, as shown on the right side of Fig. 24. If this is not the case, the calculation may be considered plausible. Otherwise, their difference was compared with the IEA’s NES energy consumption values. NES values represent industrial energy consumption that is not attributed to a specific sector, often due to underreporting or incomplete documentation of the reporting countries. A high NES value suggests that the country has reported only the energy consumption of the whole industrial sector without providing details regarding industrial subsector in which the energy was utilized. Additionally, we took a closer look into the NES share for the analysed 159 world countries, see Fig. 25. It is noticeable that a NES share of 30% up to 100% occurs for around half of the countries globally, and around 12% of the countries has a NES share of nearly 100%. However, if we observe the global NES energy amount, it turns out that countries having a NES share of up to around 25% constitute almost 90% of the global NES industrial energy demand. In other words, there are many countries that report poorly, but those countries with high energy demand seem to report well in most cases. Finally, if the difference between the total calculated energy demand and the IEA energy balance is smaller than NES energy, the discrepancy between the calculation and the IEA statistics can be explained by the NES values, confirming our calculations. If the total calculated energy demand is greater than the IEA energy balance, but the difference is higher than the NES energy a plausibility error is indicated.

The plausibility check was applied per TIAM region and summarized in Table 4. To make the difference between calculated energy demand and IEA energy balance (IEA_EB) comparable with the NES industrial energy consumption, both the calculated values and the IEA balances are summed across all five industrial subsectors. The table shows that these differences are lower than NES energy consumptions as their ratios are below 100% for all TIAM regions. This indicates that calculated final energy demand is not overestimated. The difference between the calculations and statistics is likely due to under-reporting of the energy consumption in some countries. Still, the IEA database was the most comprehensive worldwide industrial energy consumption related database the authors could find and was thus considered appropriate to use.

**Table 4:** Comparison of the calculated energy demand and energy demand statistics.

Region (see Fig. 17)	Calculated BAT Energy Demand (CED_ BAT) [PJ]	Calculated Energy Demand (CED) [PJ]	Calculation difference CED-IEA_EB [PJ]	$\frac{({\bf{CED}}-{\bf{IEA\_EB}})}{{\bf{NES}}}\,$[%]
**AFR**	1718.22	2387.10	552.57	25%
**AUS**	493.55	902.41	39.43	64%
**CAN**	1409.77	1866.24	—	—
**CHI**	37790.44	41462.71	445.02	17%
**CSA**	2674.56	3883.87	—	—
**EEU**	1161.78	1868.55	—	—
**FSU**	4530.45	9443.10	—	—
**GER**	2067.06	2582.29	0.82	1%
**IND**	5583.22	9428.01	3127.23	73%
**JPN**	3381.95	4548.44	—	—
**MEA**	4790.35	7604.94	2662.62	45%
**MEX**	580.64	933.96	147.78	19%
**ODA**	3722.34	7508.42	688.27	17%
**SKO**	2350.95	3748.13	—	—
**USA**	8025.74	11871.95	—	—
**WEU**	5657.19	7415.97	431.11	79%

## Data Availability

The data produced by this study are stored on Zenodo under 10.5281/zenodo.15524913^107^.

## Code availability

No specific custom code has been used.

## References

[1] Verdolini, E. *et al*. Industrial deep decarbonisation: modelling approaches and data challenges. Available at https://media.rff.org/documents/Report_23-10v4.pdf (2023).

[2] Ren, T., Patel, M. & Blok, K. Olefins from conventional and heavy feedstocks: Energy use in steam cracking and alternative processes. *Energy* **31**, 425–451, 10.1016/j.energy.2005.04.001 (2006).

[3] Haribal, V. P., Chen, Y., Neal, L. & Li, F. Intensification of Ethylene Production from Naphtha via a Redox Oxy-Cracking Scheme: Process Simulations and Analysis. *Engineering* **4**, 714–721, 10.1016/j.eng.2018.08.001 (2018).

[4] Saygın, D., Patel, M. K., Tam, C. & Gielen, D. J. Chemical and Petrochemical Sector. Available at https://www.iea.org/reports/chemical-and-petrochemical-sector (2009).

[5] Bazanella, A. M. & Ausfelder, F. Low carbon energy and feedstock for the European chemical industry. Available at https://dechema.de/dechema_media/Downloads/Positionspapiere/Technology_study_Low_carbon_energy_and_feedstock_for_the_European_chemical_industry.pdf (2017).

[6] IEA. The Future of Petrochemicals. Towards more sustainable plastics and fertilisers. Available at https://www.oecd.org/content/dam/oecd/en/publications/reports/2018/10/the-future-of-petrochemicals_g1g98d79/9789264307414-en.pdf (2018).

[7] IEA. Technology Roadmap - Low-Carbon Transition in the Cement Industry. Available at https://www.iea.org/reports/technology-roadmap-low-carbon-transition-in-the-cement-industry (2018).

[8] Ünlü, G. *et al*. MESSAGEix-Materials v1.1.0: representation of material flows and stocks in an integrated assessment model. *Geoscientific Model Development* **17**, 8321–8352, 10.5194/gmd-17-8321-2024 (2024).

[9] Mathiesen, B. V. *et al*. The green transition of industry – An introduction to IndustryPLAN. *Smart Energy* **11**, 100111, 10.1016/j.segy.2023.100111 (2023).

[10] Hasanbeigi, A., Arens, M., Cardenas, J. C. R., Price, L. & Triolo, R. Comparison of carbon dioxide emissions intensity of steel production in China, Germany, Mexico, and the United States. *Resources, Conservation and Recycling* **113**, 127–139, 10.1016/j.resconrec.2016.06.008 (2016).

[11] Hasanbeigi, A. *et al*. Comparison of iron and steel production energy use and energy intensity in China and the U.S. *Journal of Cleaner Production* **65**, 108–119, 10.1016/j.jclepro.2013.09.047 (2014).

[12] Wang, P. *et al*. Regional disparities in steel production and restrictions to progress on global decarbonization: A cross-national analysis. *Renewable and Sustainable Energy Reviews* **161**, 112367, 10.1016/j.rser.2022.112367 (2022).

[13] IEA. World Energy Statistics 2018. OECD Publishing. Available at 10.1787/world_energy_stats-2018-en (2018).

[14] Eurostat. Total production ds-056121. Available at https://ec.europa.eu/eurostat/databrowser/view/ds-056121/legacyMultiFreq/table?lang=en (2023).

[15] Breuning, L. & Kerekeš, A. endemo – Energy Demand Modeling for Europe. In *Energy Sciences for Europe’s Green Deal. 11th Colloquium of the Munich School of Engineering* (Garching bei München, 2021).

[16] U.S. Department of the Interior, U.S. Geological Survey. *Minerals Yearbook 2020*. 1st ed. (2023).

[17] Labriet, M., Loulou, R. & Kanudia, A. The TIMES Integrated Assessment Model (ETSAP-TIAM). Contribution to the discussion. Available at https://www.iamconsortium.org/wp-content/uploads/2020/03/090916_10_Tosato.pdf (2009).

[18] Lippkau, F., Rupakula, G. D. & Blesl, M. Emission Free Energy Carriers and the Impact of Trade to Achieve the 1.5 °C Target: A Global Perspective of Hydrogen and Ammonia. In *Aligning the Energy Transition with the Sustainable Development Goals*, edited by M. Labriet, K. Espegren, G. Giannakidis & B. Ó Gallachóir, **Vol. 101**, pp. 247–270 (Springer Nature Switzerland, Cham, 2024).

[19] Wilkinson, M. D. *et al*. The FAIR Guiding Principles for scientific data management and stewardship. *Scientific Data* **3**, 160018, 10.1038/sdata.2016.18 (2016). 26978244 10.1038/sdata.2016.18 PMC4792175

[20] Joint Research Centre: Institute for Prospective Technological Studies, Remus, R., Roudier, S., Delgado Sancho, L. & Aguado-Monsonet, M. *Best available techniques (BAT) reference document for iron and steel production – Industrial emissions Directive 2010/75/EU – Integrated pollution prevention and control* (Publications Office, 2013).

[21] U.S. Department of the Interior, U.S. Geological Survey. Iron and Steel Statistics and Information. Available at https://www.usgs.gov/centers/national-minerals-information-center/iron-and-steel-statistics-and-information (2021).

[22] Menzie, W. D. *et al*. The Global Flow of Aluminum From 2006 Through 2025. U.S. Department of the Interior; U.S. Geological Survey (2010).

[23] World bureau of metal statistics. Production of secondary aluminum in China from 2010 to 2021. Available at https://www.statista.com/statistics/1126256/china-secondary-aluminum-production-volume/ (2022).

[24] World bureau of metal statistics. Production volume of secondary aluminum in South Korea from 2011 to 2021. Available at https://www.statista.com/statistics/1129635/south-korea-secondary-aluminum-production-volume/ (2022).

[25] Hübner, T., Guminski, A., Rouyrre, E. & von Roon, S. Branchensteckbrief der NE-Metallindustrie. Available at https://www.bmwk.de/Redaktion/DE/Downloads/E/energiewende-in-der-industrie-ap2a-branchensteckbrief-metall.pdf?__blob=publicationFile&v=4 (2019).

[26] U.S. Department of the Interior, U.S. Geological Survey. Mineral Commodity Summaries. Aluminium. Available at https://pubs.usgs.gov/periodicals/mcs2020/mcs2020-aluminum.pdf (2020).

[27] Abralatas. The Future of the Aluminum Can. Available at https://www.ball.com/getmedia/18d5fe0a-6cac-49d5-98d8-b31ed6097092/Future-of-the-Aluminum-Can.pdf (2024).

[28] Moreno-Leiva, S. *et al*. Renewable energy in copper production: A review on systems design and methodological approaches. *Journal of Cleaner Production* **246**, 118978, 10.1016/j.jclepro.2019.118978 (2020).

[29] McCarten, M. *et al*. Global Database of Cement Production Assets. Available at https://www.cgfi.ac.uk/spatial-finance-initiative/geoasset-project/cement/ (2021). 10.1038/s41597-023-02599-w PMC10575953 37833339

[30] Andrew, R. Global CO2 emissions from cement production. The Cement Production dataset. Available at https://zenodo.org/records/7875557#.ZFtweHZBybh (2023).

[31] Zier, M., Stenzel, P., Kotzur, L. & Stolten, D. A review of decarbonization options for the glass industry. *Energy Conversion and Management: X* **10**, 100083, 10.1016/j.ecmx.2021.100083 (2021).

[32] U.S. Department of the Interior, U.S. Geological Survey. Soda Ash Statistics and Information. Available at https://www.usgs.gov/centers/national-minerals-information-center/soda-ash-statistics-and-information (2021).

[33] United Nations (UN). UN Commodity Trade Statistics Database. Available at https://comtradeplus.un.org/.

[34] U.S. Department of the Interior, U.S. Geological Survey. Lime Statistics and Information. Available at https://www.usgs.gov/centers/national-minerals-information-center/lime-statistics-and-information (2021).

[35] Food and Agriculture Orgization of the United Nations. Forestry Production and Trade. Available at https://www.fao.org/faostat/en/#data/FO (2023).

[36] Chatterton, C. Methanol as a vessel fuel & energy carrier. Available at https://www.methanol.org/wp-content/uploads/2019/09/Methanol-as-a-vessel-fuel-and-energy-carrier.pdf (2019).

[37] Statista Research Department. Methanol production in the United States from 1990 to 2019. Available at https://www.statista.com/statistics/974802/us-methanol-production-volume/ (2023).

[38] Verband der Chemischen Industrie e. V. Chemiewirtschaft in Zahlen. Available at https://www.vci.de/vci/downloads-vci/publikation/chiz-historisch/chemiewirtschaft-in-zahlen-2022.pdf (2022).

[39] Vallette, J. Chlorine and Building Materials: A Global Inventory of Production Technologies, Markets and Pollution. Phase 1: Africa, The Americas and Europe. Available at https://asbp.org.uk/wp-content/uploads/2018/08/Chlorine-Building-Materials-Phase-1-v2.pdf (2018).

[40] Vallette, J. Chlorine and Building Materials: A Global Inventory of Production Technologies, Markets and Pollution. Phase 2: Asia, 10.13140/RG.2.2.16731.62244 (2019).

[41] AgileIntel Research, Statista. Market volume of chlorine worldwide from 2015 to 2022, with a forecast for 2023 to 2030. Available at https://www.statista.com/statistics/1310477/chlorine-market-volume-worldwide/ (2023).

[42] Statista Research Department. Chlorine production in the United States from 1990 to 2019. Available at https://www.statista.com/statistics/974614/us-chlorine-production-volume/ (2020).

[43] China Chlor Alkali Industry Association. Analysis of High Quality Development of China’s Chlor-Alkali Industry. Available at https://eurochlor2025.org/wp-content/uploads/2025/05/TSEM-25-561-The-Current-Situation-of-Sustainable-Development-in-China-Z.-Peichao.pdf (2025).

[44] IRENA. Renewable ammonia. Innovation outlook. Available at https://www.irena.org/publications/2022/May/Innovation-Outlook-Renewable-Ammonia (2022).

[45] U.S. Department of the Interior, U.S. Geological Survey. Nitrogen Statistics and Information. Available at https://www.usgs.gov/centers/national-minerals-information-center/nitrogen-statistics-and-information (2021).

[46] GlobalData. Production capacity of ethylene worldwide from 2018 to 2022. Available at https://www.statista.com/statistics/1067372/global-ethylene-production-capacity/ (2023).

[47] GlobalData. Production capacity of propylene worldwide in 2018 and 2022, with a forecast to 2030. Available at https://www.statista.com/statistics/1065879/global-propylene-production-capacity/ (2023).

[48] GlobalData. Production capacity of butadiene worldwide from 2024 to 2028. Available at https://www.statista.com/statistics/1067436/global-butadiene-production-capacity/ (2024).

[49] Seddon, D. *Petrochemical Economics: Technology Selection in a Carbon Constrained World* (Imperial College Press, 2010).

[50] American Chemistry Council. Ethylene production in the United States from 1990 to 2019. Available at https://www.statista.com/statistics/974766/us-ethylene-production-volume/ (2020).

[51] Russian Federal State Statistics Service. Россия в цифра (Russia in figures). Available at https://rosstat.gov.ru/free_doc/doc_2019/rusfig/rus19.pdf (2019).

[52] Monai, M., Gambino, M., Wannakao, S. & Weckhuysen, B. M. Propane to olefins tandem catalysis: a selective route towards light olefins production. *Chemical Society Reviews* **50**, 11503–11529, 10.1039/D1CS00357G (2021). 34661210 10.1039/d1cs00357g

[53] Bender, M. An Overview of Industrial Processes for the Production of Olefins – C4 Hydrocarbons. *ChemBioEng Reviews* **1**, 10.1002/cben.201400016 (2014).

[54] Falcke, H. *et al*. *Best Available Techniques (BAT) Reference Document for the Production of Large Volume Organic Chemicals. EUR 28882 EN*. JRC109279 (Publications Office of the European Union, Luxembourg, 2017).

[55] Plastindia Foundation. Plastics Industry Status Report – India – 2021-22 & 1H 2022-23 Update. Available at https://icpe.in/pdf/Plastics%20Industry%20Status%20%20Report-India%20-2021-22.pdf (2023).

[56] Center for Strategic Research. Нефтегазохимия в России: возможности для роста (Petrochemistry in Russia: opportunities for growth). Available at https://www.csr.ru/upload/iblock/d88/9vy10zbpvss8f0h8z31616dij5zab3s6.pdf (2021).

[57] American Chemistry Council. Propylene production in the United States from 1990 to 2019. Available at https://www.statista.com/statistics/974833/us-propylene-production-volume/ (2020).

[58] statista. Ethylene demand and production capacity worldwide from 2015 to 2022. Available at https://www.statista.com/statistics/1246694/ethylene-demand-capacity-forecast-worldwide/ (2020).

[59] statista. Propylene demand and capacity worldwide from 2015 to 2022. Available at https://www.statista.com/statistics/1246689/propylene-demand-capacity-forecast-worldwide/ (2020).

[60] Bender, M. Global Aromatics Supply - Today and Tomorrow. *Oil Gas European Magazine* **39**, 209–212 (2013).

[61] Rompetrol Rafinare S.A. Raport anual 2005. Available at https://rompetrol-rafinare.kmginternational.com/upload/files/raport_anual_2005_221.pdf (2006).

[62] Nexant. PERP Report: Benzene/Toluene 06/07-6. Available at https://pdfcoffee.com/135-perp0607-61-benzene-toluene-nexant-pdf-free.html (2007).

[63] KOSIS. Production volume of toluene in South Korea from 2013 to 2022. Available at https://www.statista.com/statistics/732204/south-korea-toluene-production-volume/ (2023).

[64] KOSIS. Production volume of benzene in South Korea from 2013 to 2023. Available at https://www.statista.com/statistics/732199/south-korea-benzene-production-volume/ (2024).

[65] American Chemistry Council. Toluene production in the United States from 1990 to 2019. Available at https://www.statista.com/statistics/974854/us-toluene-production-volume/ (2020).

[66] American Chemistry Council. Benzene production in the United States from 1990 to 2019. Available at https://www.statista.com/statistics/974691/us-benzene-production-volume/ (2020).

[67] American Chemistry Council. p-Xylene production in the United States from 1990 to 2019. Available at https://www.statista.com/statistics/975537/us-p-xylene-production-volume/.

[68] METI. Yearbook of current production statistics. Chemical Industry. Available at https://www.meti.go.jp/statistics/tyo/seidou/result/gaiyo/resourceData/02_kagaku/nenpo/h2dbb2018k.pdf (2018).

[69] Russian Federal State Statistics Service. Production of main types of products in physical terms (annual data since 2017 - in accordance with OKPD2). Available at https://rosstat.gov.ru/storage/mediabank/Proizvodstvo_god_s_2017.xlsx (2024).

[70] ThyssenKrupp Industrial Solutions AG. World Market Leader in Aromatics Extraction. Available at https://ucpcdn.thyssenkrupp.com/_legacy/UCPthyssenkruppBAIS/assets.files/products___services/chemical_plants___processes/tkis_aromatics.pdf (2015).

[71] AgileIntel Research. Market volume of benzene worldwide from 2015 to 2022, with a forecast for 2023 to 2030. Available at https://www.statista.com/statistics/1245172/benzene-market-volume-worldwide/ (2023).

[72] AgileIntel Research. Market volume of toluene worldwide from 2015 to 2022, with a forecast for 2023 to 2030. Available at https://www.statista.com/statistics/1245224/toluene-market-volume-worldwide/ (2023).

[73] Bloomberg, Krungsri Research & Statista estimates. Xylene demand and production capacity worldwide from 2015 to 2022. Available at https://www.statista.com/statistics/1246700/xylene-demand-capacity-forecast-worldwide/ (2020).

[74] World Steel Association. Steel industry co-products. Fact sheet. Available at https://worldsteel.org/wp-content/uploads/Fact-sheet-Steel-industry-co-products.pdf (2021).

[75] Wörtler, M. *et al*. Steel´s Contribution to a Low-Carbon Europe 2050. Available at https://www.wvstahl.de/wp-content/uploads/Schlussbericht-Studie-Low-carbon-Europe-2050_-Mai-20131.pdf (2013).

[76] Pardo, N. & Moya, J. A. Prospective scenarios on energy efficiency and CO2 emissions in the European Iron & Steel industry. *Energy* **54**, 113–128, 10.1016/j.energy.2013.03.015 (2013).

[77] Otto, A. *et al*. Power-to-Steel: Reducing CO2 through the Integration of Renewable Energy and Hydrogen into the German Steel Industry. *Energies* **10**, 451, 10.3390/en10040451 (2017).

[78] Fruehan, R. J., Fortini, O., Paxton, H. W. & Brindle, R. Theoretical minimum energies to produce steel for selected conditions. Available at http://large.stanford.edu/courses/2016/ph240/martelaro1/docs/fruehan-mar00.pdf (2000).

[79] Cusano G. *et al*. Best Available Techniques (BAT) Reference Document for the Non-Ferrous Metals Industries. Industrial Emissions Directive 2010/75/EU (Integrated Pollution Prevention and Control). *Technical guidance*. *1831-9424*; 10.2760/8224.

[80] Kuder, R. Energieeffizienz in der Industrie. Modellgestützte Analyse des effizienten Energieeinsatzes in der EU-27 mit Fokus auf den Industriesektor. Dissertation. Available at https://d-nb.info/1049260554/34 (2014).

[81] U.S. Department of Energy. U.S. Energy Requirements for Aluminum Production. Historical Perspective, Theoretical Limits and Current Practices. Available at https://www1.eere.energy.gov/manufacturing/resources/aluminum/pdfs/al_theoretical.pdf (2007).

[82] Allen, M. Mining energy consumption 2021. Available at https://www.ceecthefuture.org/resources/mining-energy-consumption-2021 (2021).

[83] Alvarado, S., Maldonado, P. & Jaques, I. Energy and environmental implications of copper production. *Energy* **24**, 307–316, 10.1016/S0360-5442(98)00093-0 (1999).

[84] Schifo, J. F. & Radia, J. T. Theoretical/Best Practice Energy Use In Metalcasting Operations. Available at https://www.energy.gov/sites/prod/files/2013/11/f4/doebestpractice_052804.pdf (2004).

[85] Beukes, N. T. & Badenhorst, J. Copper electrowinning: theoretical and practical design. *The Journal of The Southern African Institute of Mining and Metallurgy*, 343–356 (2009).

[86] Madlool, N. A., Saidur, R., Hossain, M. S. & Rahim, N. A. A critical review on energy use and savings in the cement industries. *Renewable and Sustainable Energy Reviews* **15**, 2042–2060, 10.1016/j.rser.2011.01.005 (2011).

[87] European Cement Research Academy. Development of State of the Art-Techniques in Cement Manufacturing: Trying to Look Ahead, Revision 2017. Available at https://docs.wbcsd.org/2017/06/CSI_ECRA_Technology_Papers_2017.pdf (2017).

[88] Worrell, E., Kermeli, K. & Galitsky, C. Energy Efficiency Improvement and Cost Saving Opportunities for Cement Making. Available at https://www.energystar.gov/sites/default/files/tools/ENERGY%20STAR%20Guide%20for%20the%20Cement%20Industry%2027_08_2013_Rev%20js%20reformat%2011192014.pdf (2013).

[89] glassglobal Group. Facts and Figures. Available at https://plants.glassglobal.com/.

[90] Barón, C., Perpiñán, J., Bailera, M. & Peña, B. Techno-economic assessment of glassmaking decarbonization through integration of calcium looping carbon capture and power-to-gas technologies. *Sustainable Production and Consumption* **41**, 121–133, 10.1016/j.spc.2023.07.029 (2023).

[91] Stork M., Meindertsma W., Overgaag M. & Maarten N. A competitive and efficient lime industry - Technical report. Available at https://www.eula.eu/wp-content/uploads/2019/02/A-Competitive-and-Efficient-Lime-Industry-Technical-report-by-Ecofys_0.pdf (2014).

[92] International Energy Agency & Organisation for Economic Co-operation and Development. Energy technology transitions for industry. Strategies for the next industrial revolution. Available at 10.1787/9789264068612-en (2009).

[93] Miller, T., Kramer, C. & Fisher, A. Bandwidth study on energy use and potential energy saving opportunities in U.S. pulp and paper manufacturing. Available at https://www.energy.gov/sites/prod/files/2015/08/f26/pulp_and_paper_bandwidth_report.pdf (2015).

[94] EuroChlor. The electrolysis process and its thermodynamic limits. Available at https://www.eurochlor.org/wp-content/uploads/2021/04/11-Electrolysis-thermodynamics.pdf (2018).

[95] Ausfelder, F. *et al*. Roadmap Chemie 2050 Deutschland. Auf dem Weg zu einer treibhausgasneutralen chemischen Industrie in. Available at https://dechema.de/chemie2050-path-123211,124930.html (2019).

[96] Kullmann, F. Recycling- und Defossilisierungsmaßnahmen der energieintensiven Industrie Deutschlands im Kontext von CO2-Reduktionsstrategien. Available at https://juser.fz-juelich.de/record/917475/files/Energie_Umwelt_598.pdf (2022).

[97] IEA. Ammonia Technology Roadmap. Towards more sustainable nitrogen fertiliser production; 10.1787/f6daa4a0-en (2021).

[98] Rouwenhorst, K. H., van der Ham, A. G., Mul, G. & Kersten, S. R. Islanded ammonia power systems: Technology review & conceptual process design. *Renewable and Sustainable Energy Reviews* **114**, 109339, 10.1016/j.rser.2019.109339 (2019).

[99] Worrell, E., Price, L., Neelis, M., Galitsky, C. & Zhou, N. World best practice energy intensity values for selected industrial sectors; 10.2172/927032 (2007).

[100] Ren, T., Patel, M. K. & Blok, K. Steam cracking and methane to olefins: Energy use, CO2 emissions and production costs. *Energy* **33**, 817–833, 10.1016/j.energy.2008.01.002 (2008).

[101] Richardson, J. Global oversupply of petrochemicals to hit 218m tonnes in 2023 - the highest in any other year since 1990. Available at https://www.icis.com/asian-chemical-connections/2023/03/global-oversupply-of-petrochemicals-to-hit-218m-tonnes-in-2023-the-highest-in-any-year-since-1990/ (2023).

[102] Gogate, M. R. Methanol-to-olefins process technology: current status and future prospects. *Petroleum Science and Technology* **37**, 559–565, 10.1080/10916466.2018.1555589 (2019).

[103] Bolson, N., Cullen, L. & Cullen, J. A robust framework for estimating theoretical minimum energy requirements for industrial processes. *Energy* **322**, 135411, 10.1016/j.energy.2025.135411 (2025).

[104] IHS Chemical. Naphtha Catalytic Cracking. Available at https://cdn.ihs.com/www/pdf/RP29K-toc.pdf (2017).

[105] Brueske, S., Kramer, C. & Fisher, A. Bandwidth study on energy use and potential energy saving opportunities in U.S. chemical manufacturing. Available at https://www.energy.gov/sites/prod/files/2015/08/f26/chemical_bandwidth_report.pdf (2015).

[106] KOSIS. Production volume of aluminum plates in South Korea from 2013 to 2023. Available at https://www.statista.com/statistics/733050/south-korea-aluminium-plate-and-strip-production-volume/ (2024).

[107] Kerekeš, A. *et al*. Follow-ETSAP/Industrial-energy-demands: Industrial Energy Demand Data Set - v2.0.2; 10.5281/zenodo.15524913 (2025).

[108] Bureau of Energy Efficiency, Government of India. Normalization document and monitoring & verification guidelines. Aluminium Sector. Available at https://beeindia.gov.in/sites/default/files/Aluminium-1-44.pdf (2015).

[109] Kumar Banerjee, P. Sustainability of the Indian Aluminium Industry: Challenges and Opportunities. In *Proceedings of 35th International ICSOBA Conference*. Available at https://icsoba.org/assets/files/publications/2017/KN05S%20-%20Sustainability%20of%20the%20Indian%20Aluminium%20Industry%20-%20Challenges%20and%20Opportunities.pdf (2017).

[110] Emirates Global Aluminium (EGA). EGA 2021 Sustainability Report. Available at https://www.ega.ae/en/sustainability/sustainability-reports/ (2021).

